# Using wearable technology to evaluate the electrodermal activity of therapists assessing challenging behavior

**DOI:** 10.1002/jaba.70050

**Published:** 2026-01-23

**Authors:** Emily K. Sullivan, Tara A. Fahmie, Jamie E. Gehringer

**Affiliations:** ^1^ Integrated Center for Autism Spectrum Disorders University of Nebraska Medical Center's Munroe‐Meyer Institute Omaha NE USA; ^2^ Severe Behavior Department University of Nebraska Medical Center's Munroe‐Meyer Institute Omaha NE USA; ^3^ Physical Therapy Department University of Nebraska Medical Center's Munroe‐Meyer Institute Omaha NE USA

**Keywords:** electrodermal activity, functional analysis, physiological arousal, skin conductance, therapist well‐being

## Abstract

Identifying objective ways to measure a therapist's physiological responding when encountering challenging behavior has the potential to guide future work in staff performance, well‐being, and retention. The current technical report summarizes controlled measures of therapists' electrodermal activity (EDA) while implementing functional analyses of challenging behavior. The technology used to monitor EDA, analyses relevant to EDA in the context of challenging behavior, and technical barriers related to the use of these measures are discussed. Preliminary data from three therapists suggested that indicators of acute physiological arousal are present in functional analyses, particularly surrounding occurrences of challenging behavior. Support for the further development of these technologies is provided.

Individuals diagnosed with intellectual and developmental disabilities face an increased risk of displaying some form of challenging behavior relative to their neurotypical peers (Gurney et al., 2006; McClintock et al., 2003). Broadly, the term challenging behavior refers to responses that have the potential to harm the individual exhibiting them, others, or property (e.g., self‐injury, aggression, and destruction of property). Exhibiting challenging behavior not only poses substantial safety risks but can also reduce access to educational and social opportunities (Nicholls et al., 2019) and decrease the overall well‐being of the individual and their caregivers (Chiang & Wineman, 2014; Demirpençe Seçinti et al., 2024; Levy et al., 2023; Rodriguez et al., 2019). Mirroring reports from familial caregivers of children with challenging behavior (Postorino et al., 2019), therapist exposure to challenging behavior has also been associated with increased stress (Hensel et al., 2015; Mills & Rose, 2011).

Efforts to ensure the safety of therapists and clients with challenging behavior have been a high priority for decades and remain a central focus of service delivery. For example, several studies suggest ways to gauge risk and maximize safety when assessing challenging behavior in a functional analysis (e.g., Deochand et al., 2020; Frank‐Crawford et al., 2024; Schroeder et al., 2024). Additionally, a growing body of research has evaluated the role of protective equipment (e.g., arm guards, headgear, compression sleeves; Daraiseh et al., 2018; Hardesty et al., 2025; Irwin Helvey et al., 2024; Sullivan, Zarcone, et al., 2025) in decreasing therapist injury when encountering challenging behavior. However, measures of therapist responses to challenging behavior have largely been overt in nature, leaving the physiological effects of challenging behavior unknown (Sullivan, Narducci, et al., 2025). Insofar as challenging behavior is classified as a potential *stressor* (i.e., a stimulus that disrupts physical or psychological homeostasis; Chu et al., 2024), the physiological changes that occur in the presence of challenging behavior warrant further investigation.

Electrodermal activity (EDA) is widely considered among the most valid and sensitive physiological markers of a *stress response*, characterized as an adaptive or maladaptive state of arousal (Christopoulos et al., 2019; Kyriakou et al., 2019). Previous research has measured the EDA of employees during various job‐related activities including assembly tasks (Kosch et al., 2019), immediate feedback delivery (Horvers et al., 2024), and simulated medical scenarios (Claverie et al., 2020) as well as across different workplace settings including academic institutions (Bolliger et al., 2020), intensive care hospital units (Ahmadi et al., 2022), and offices (Suni Lopez et al., 2019). Studies like these recognize that intense, repeated, or prolonged exposure to job‐related stressors may lead to both physical and mental health concerns and contribute to burnout. On the other hand, stress responses can be adaptive, as increases in arousal can physiologically prepare the body to manage internal and external challenges (Crosswell & Lockwood, 2020). For example, a stress response may accompany a heightened focus and quick responding to minimize safety threats. When managing challenging behavior, for example, a stress response may facilitate the therapist's efforts to evade aggressive attempts more quickly or more effectively block self‐injury.

Organizational researchers have suggested that evaluating EDA may assist with identifying common stressors employees encounter and in developing targeted, function‐based interventions to promote resiliency (Christopoulos et al., 2019). Such a strategy may be an even more pertinent initiative in workplaces in which employees are subjected to prolonged exposure to physical safety risks (e.g., therapists regularly interacting with individuals with challenging behavior). Intense, repeated, or prolonged exposure to stressors can lead to both physical and mental health concerns (Chu et al., 2024) and might contribute to the burnout reported by many behavioral therapists in surveys (Bottini et al., 2025; Deling et al., 2024). Similar strategies may also benefit familial caregivers of individuals with challenging behavior, who experience prolonged exposure to these same stressors at home and report similar concerns regarding their mental and physical well‐being (Machado et al., 2016; Schnabel et al., 2020; Vaz et al., 2021). Although the physiological stress responses of caregivers and therapists who regularly interact with individuals with challenging behavior remain underexplored, EDA offers a promising avenue for their evaluation.

Statistical features of EDA (i.e., minimum, maximum, and mean) have been shown to predict stress responses within specified time windows (Hosseini et al., 2022). This is because EDA captures the two major systems activated during a stress response—the autonomic nervous system and hypothalamic–pituitary–adrenal axis—which respond to regulate the activation elicited by a stressor and return responding to a steady state (Kyriakou et al., 2019). The EDA signal is commonly parsed into two components from which researchers extract features used to quantify stress. The first includes the fast‐moving changes in sympathetic nervous system arousal, captured by the phasic activity (Braithwaite et al., 2013; Zangróniz et al., 2017). The second includes the slower moving changes in autonomic system arousal, captured by the tonic activity. Among the most common features extracted from the phasic component of the EDA signal is the skin conductance response (SCR). Although SCRs vary across individuals due to interindividual factors and their occurrence may not be associated with an identifiable eliciting stimulus (nonspecific SCR; Setz et al., 2010), increases in SCRs have been shown to correlate with self‐reports of stress (Meijer et al., 2023) and are sensitive to experimental conditions designed to induce a stress reaction (van der Mee et al., 2021). When SCRs are measured in relation to exposures to programmed stimuli, these responses can provide insight into the stress elicited by specific events (event‐related SCRs).

Researchers have noted that commercially available technologies for measuring EDA (e.g., Empatica E4: Empatica, Inc., 2023; EmotiBit: Montgomery et al., 2023; see Tronstad et al., 2022, for additional examples) offer exciting research opportunities to advance insight into covert experiences (Tronstad et al., 2022). That said, further research is needed to overcome logistical challenges in ambulatory settings that may decrease data quality, such as reduced oversight of proper connection and uncontrolled participant movement (Tronstad et al., 2022). Although wearable technologies have recently made their way into the assessment of challenging behavior to automate the detection of otherwise subtle or covert client behavior—for example to evaluate the predictive validity of heart rate for client challenging behavior (McCabe & Greer, 2023) or to develop a machine‐learning framework designed to predict challenging behavior based on multiple physiological response measures (Zheng et al., 2021)—to our knowledge, the technology has not yet been extended to therapists regularly exposed to challenging behavior.

Therefore, the current technical report provides an example of the instrumentation, analysis, and procedures that may be used to monitor the EDA of therapists who encounter client challenging behavior. Additionally, we sought to evaluate the feasibility of using wearable technology for this purpose. Thus, we conducted a preliminary evaluation of these measures under test and control conditions of a functional analysis in which challenging behavior was predicted to be evoked and abated, respectively.

## METHOD

### Participants and setting

The participants^1^ of this study included three therapist–client dyads. The therapists were employed by a university outpatient clinic, and the clients were referred to the clinic for the assessment and treatment of their challenging behavior. Client inclusion criteria were (a) referral for exhibiting topographies of challenging behavior that resulted in injury to others; (b) Destructive Behavior Severity Scale^2^ ratings completed upon intake by a licensed psychologist that did not exceed a 3, which indicates that the most severe injuries resulting from challenging behavior included shallow cuts, minor scratches, or substantial swelling (Fisher et al., 2013, 2022); and (c) prior to this study, a functional analysis was completed in the outpatient clinic identifying a social function of challenging behavior (hereafter referred to as the, “prestudy functional analysis”).

All clients experienced a prestudy multielement functional analysis, which began with exposure to multiple isolated test conditions (i.e., escape, attention, tangible, and alone or no interaction) and an omnibus control condition. Any deviations deemed clinically necessary were directed by each client's clinical team (e.g., extending session length, synthesizing test conditions) until the variables controlling the occurrence of challenging behavior were identified. Clients identified as exhibiting socially mediated challenging behavior were selected for this study to increase the likelihood that challenging behavior could be reliably evoked and reduced across test and control conditions, respectively. We did not seek consent for clients to participate in this study, as the university‐affiliated institutional review board advised that the procedures reflected typical service provision in the clinic (i.e., the functional analysis in this study served as a baseline to evaluate subsequent client‐specific interventions). However, consent for the dissemination of data collected during ongoing services was received from all clients' caregivers as part of standard clinic intake procedures.

Therapists were enrolled because they were assigned as therapists to the first three clients who met the inclusion criteria and provided informed consent to participate in the study. All therapists had completed an Association for Behavior Analysis International–accredited program in applied behavior analysis prior to employment. Therapists 1 and 3 completed undergraduate programs, and Therapist 2 completed a master's program. During the study, Therapist 1 was enrolled in a master's program in behavior analysis and Therapist 2 was enrolled in an Association for Behavior Analysis International–accredited doctoral program. All therapists had previous experience working with clients who exhibited challenging behavior and implementing functional analysis procedures. As part of the clinic's standard onboarding process, all therapists received training on functional analysis procedures, which included (a) completing online courses focused on functional assessment (approximately 3 hr of content), (b) observing functional analysis sessions in the clinic, and (c) implementing the procedures in vivo with feedback from supervisors. The authors' university‐affiliated institutional review board approved the study procedures.

Dyad 1 included Client 1, a 7‐year‐old Black male with diagnoses of autism spectrum disorder; attention‐deficit/hyperactivity disorder; unspecified intellectual disability; pica; and other specified disruptive, impulse‐control, and conduct disorder. He was referred to the clinic for the assessment and treatment of aggression, disruption, elopement, and pica. Ratings on the Destructive Behavior Severity Scale for these topographies ranged from 1 to 3. Client 1 used a speech‐generating device and gestures to communicate. His height and weight were reported to be within typical age limits, and he attended the clinic for 3 hr per day, 5 days per week. Therapist 1 was a 24‐year‐old White male assigned to Client 1's case and had been employed by the clinic for approximately 2 years and 4 months before the study.

Dyad 2 included Client 2, an 11‐year‐old Hispanic/Latina female with diagnoses of other specified disruptive, impulse‐control, and conduct disorder; autoimmune encephalitis; and psychosis. She was referred to the clinic for the assessment and treatment of aggression, disruption, and property destruction. Ratings on the Destructive Behavior Severity Scale for these topographies ranged from 1 to 3. She communicated vocally using short sentences, in both English and Spanish. Her height and weight were reported to be within age‐typical limits, and she attended the clinic for 6 hr per day, 5 days per week. Therapist 2 was a 24‐year‐old White female assigned to Client 2's case and had been employed by the clinic for approximately 1.5 months before the study.

Dyad 3 included Client 3, a 12‐year‐old White male with diagnoses of autism spectrum disorder; other specified disruptive, impulse‐control, and conduct disorder; stereotypic movement disorder with self‐injurious behavior (mild); and unspecified intellectual disability. He was referred to the clinic for the assessment and treatment of aggression and self‐injury. Ratings on the Destructive Behavior Severity Scale for these challenging behavior topographies ranged from 2 to 3, and his height and weight were within age‐typical limits. He communicated vocally in English via conversations and attended the clinic 6 hr per day, 5 days per week. Therapist 3 was a 24‐year‐old female (of unknown race and ethnicity) assigned to Client 3's case and had been employed by the clinic for approximately 3 months before the study.

All sessions were conducted in therapy rooms at the outpatient clinic. Each room measured approximately 3.7 × 3 m and had padding on the walls and floor, a two‐way intercom, and a one‐way observation mirror. Materials relevant to client‐specific functional analysis sessions (e.g., work materials, preferred tangible items, a table, and chairs) were present in the therapy rooms.

### Apparatus and response measurement

#### Empatica E4 wristband

The Empatica E4 is a wearable wristband device designed for continuous real‐time acquisition of physiological data in ambulatory settings (Borrego et al., 2019; Empatica, Inc., 2023; McCarthy et al., 2016). It incorporates multiple sensors to capture a range of psychophysiological measures. Specific to our study, the Empatica E4 recorded EDA by measuring changes in skin conductance, which reflect sweat gland activity produced by sympathetic nervous system arousal, at a sampling frequency of 4 Hz (i.e., the device recorded 4 EDA samples per second). The Empatica E4 also records additional measures not included in this study (e.g., photoplethysmography, blood volume pulse, heart rate, and heart rate variability; Empatica, Inc., 2023) and includes a three‐axis accelerometer that captures movement data, providing information on physical activity levels and body position. Finally, the Empatica E4 uses an infrared thermopile sensor to measure peripheral skin temperature. Together, these measures provide valuable insights into physiological responses related to stress and physical activity in ambulatory environments (Hosseini et al., 2022).

#### Cometrics

Observational and physiological data were synchronized and recorded using *cometrics* (Arce, 2023; Arce et al., 2023; Phipps et al., 2024), a free and open‐source software distributed under the permissive MIT license, which facilitates unrestricted use, modification, and distribution provided copyright and license attribution are maintained. Item A of the Supporting Information depicts the cometrics platform display. Designed by a multidisciplinary team of behavior analysts and computer engineers, this clinical tool enables users to collect, record, and evaluate frequency and duration‐based target responses, physiological signals, and video data simultaneously. The software's Python 3.8 source code is available in its GitHub repository (see Arce, 2023), and instructions are located in the “Getting Started” section. For broad accessibility, a past release of *cometrics* is available in the Microsoft Store, offering streamlined installation and automatic updates to mitigate institutional firewall issues. More recent versions are available, with precompiled binaries in the GitHub Releases section, allowing direct installation via the Microsoft Software Installer. Developers can obtain the complete codebase by cloning the GitHub repository, enabling direct engagement with the Python 3.8 development environment.

#### Response measurement and interobserver agreement

Primary data collectors used *cometrics* to collect data on challenging behavior and therapist EDA for each session (Arce et al., 2023). Recording these responses simultaneously enabled examination of the relative changes in therapist EDA in relation to instances of challenging behavior. Secondary independent data collectors used DataPal 1.0 to record each occurrence of the client's challenging behavior and session duration using the same keystroke file as cometrics. All sessions were video recorded to allow secondary data collectors to collect data from the session videos retrospectively.

#### Challenging behavior

Client challenging behavior included any response topographies in the response class reinforced in the prestudy functional analyses that had the potential to result in contact with the therapist. Client 1's challenging behavior was defined as any instance of scratching, pinching, hitting, or grabbing others, and throwing items. Client 2's challenging behavior was defined as any instance of hitting, punching, or biting others; hand‐to‐head self‐injury; and throwing items. Hand‐to‐head self‐injury was included for Client 2 because blocking responses regularly resulted in therapist contact during the prestudy functional analysis. Client 3's challenging behavior was defined as hitting, punching, grabbing, or biting others; hand‐to‐head self‐injury; and throwing items.

#### Electrodermal activity

Therapist EDA was the physiological measure recorded using the Empatica E4 wristband, and features of the extracted signal components were used to quantify each therapist's physiological stress during sessions. The two primary measures included phasic and tonic activity. The phasic activity captured the fast‐moving components of the signal, indicating peak conductivity and increased sympathetic nervous system arousal (Braithwaite et al., 2013). The tonic activity consisted of slow variations in the signal, indicating general changes in autonomic nervous system arousal or skin conductance level (Braithwaite et al., 2013). Previous research has suggested that changes in the amplitude and frequency of both phasic and tonic components of the EDA signal covary with different emotional states (e.g., Veeranki et al., 2024; Zangróniz et al., 2017). For example, greater phasic and tonic EDA relative to baseline readings indicates heightened arousal, which suggests that the individual is experiencing acute stress, such as increased anxiety or heightened focus. In contrast, lower arousal relative to baseline suggests that the individual is experiencing lower valence states, such as relaxation, calmness, or boredom. Thus, for the purposes of this study, we conceptualized greater amplitudes of the EDA signal components and increased frequency of the extracted features as indicators that the therapists were experiencing increased physiological arousal.

The raw EDA output was processed using open‐source software, including *NeuroKit2* (Makowski et al., 2021) in Python 3 (Van Rossum & Drake, 2009) with Visual Studio Code, and the MATLAB analysis software Ledalab V3.4.9 (Benedek & Kaernbach, 2010). Additional processing and extraction details are described in the procedures. Measures produced using *NeuroKit2* included the cleaned (i.e., processed) EDA signal and the extracted phasic and tonic components. Measures produced using Ledalab V3.4.9 included extracted features of the EDA signal. The threshold set for detecting and quantifying a significant SCR was an amplitude of 0.05 microsiemens (μS; Posada‐Quintero & Chon, 2020). That is, only the rapid changes in phasic conductivity at or above an amplitude of 0.05 μS were quantified as SCRs. Additional measures specific to event‐related SCRs included the number of significant SCRs and the mean skin conductance value within the 1–5‐s response window following the eliciting event (i.e., challenging behavior).

We calculated the conditional probability of an event‐related SCR following an instance of challenging behavior and the unconditional probability of an SCR (nonspecific or event‐related) occurring at any point across all sessions. Each instance of challenging behavior and SCR lasted 1 s. We divided the total number of event‐related SCRs by the total occurrences of challenging behavior across all sessions to calculate the conditional probability of an event‐related SCR given challenging behavior (Fritz et al., 2013). To calculate the unconditional probability of SCRs, we divided the total number of SCRs during all sessions by the number of opportunities for an SCR to occur (i.e., each second of all sessions was quantified as an opportunity during which an SCR could occur; Borrero & Borrero, 2008). Any duration of sessions corresponding to EDA data loss was excluded from this calculation (described in detail in the results).

### Interobserver agreement and procedural fidelity

Independent secondary data collectors used laptop computers running the real‐time data collection software DataPal 1.0 (i.e., a beta version of BDataPro; Bullock et al., 2017) to collect data on challenging behavior in vivo or retrospectively from session video recordings. DataPal 1.0 was selected because this software was typically used by clinic employees during service delivery and the keystroke files and data collected were compatible with those generated in *cometrics* (Arce et al., 2023). This improved the feasibility of independent data collection throughout the study while maintaining the integrity of interobserver agreement calculated across the compatible platforms. A second observer independently scored at least 30% of sessions (33, 66.67, and 67.67%, respectively) for the three dyads to assess interobserver agreement on client challenging behavior measures. The mean count per interval was calculated by dividing the total number of 10‐s intervals with the same frequency by the total number of intervals and multiplying by 100%. Interobserver agreement was 98.61% (range: 97.50%–100%) for Dyad 1, 95.56% (range: 92.50%–97.50%) for Dyad 2, and 100% for Dyad 3. Interobserver agreement for physiological measures was not assessed.

Procedural fidelity of the functional analysis test condition implementation was assessed by scoring whether the therapist withheld the putative reinforcers at the beginning of the session, delivered the reinforcers contingent on each occurrence of challenging behavior, and removed the reinforcers following 30 s of access, in alignment with the research protocol. For control sessions, procedural fidelity was assessed by scoring the therapist's noncontingent delivery of reinforcers. An observer scored procedural fidelity for 100% of sessions for the three therapists, and scores were 91.67% (range: 50%–100%), 100%, and 100%, respectively.

### Procedure

#### Functional analysis sessions

Our dependent measures were examined within the context of a functional analysis to increase the likelihood of exposure to conditions with and without the occurrence of challenging behavior. Each therapist wore an Empatica E4 wristband to measure their EDA in real‐time during all functional analysis sessions and one baseline session. The test and control conditions evaluated in this study replicated those implemented in each client's prestudy functional analysis, using a sequential test–control or a pairwise design (Iwata et al., 1994). The test sessions employed the contingency identified to maintain (or evoke the highest rate of) challenging behavior in each client's prestudy functional analysis. Client 1's test condition included an escape to access attention contingency, Client 2's included an escape to access mand compliance contingency, and Client 3's included an escape to access vocal stereotypy contingency. Sessions were 10 min for Clients 1 and 3 and 5 min for Client 2. Each test session began with the therapist restricting access to the putative reinforcers and thereafter providing access for 30 s contingent on each occurrence of challenging behavior. Each control session involved the therapist providing continuous, noncontingent access to attention, tangibles, and escape from demands, with no programmed consequences for challenging behavior. The functional analysis consisted of three control–test series (a total of six sessions), beginning with a control session.

Baseline recordings of therapist EDA were conducted in the same therapy room as functional analysis sessions, without relevant session materials or clients present. Baseline session durations matched those of the functional analysis sessions for the client within the corresponding dyad. Therapists were instructed to behave as they typically would and were informed that they would receive a signal (i.e., knock on the one‐way mirror) at the start and end of the session. No additional instructions were provided. All therapists chose to sit in a chair at a table in the therapy room for the entire baseline session.

#### Electrodermal activity extraction and processing

Following each session, data collected using *cometrics* was exported in .json format. We used the open‐source MATLAB analysis software to convert the cometrics.json file into a Microsoft Excel format (.xlsx), which contained data on each occurrence of challenging behavior aligned in time with the EDA samples collected, as they were simultaneously collected across the session. See Supporting Information Item B for the MATLAB code used to transform the output. The MATLAB .xlsx file was then converted into a .csv format compatible with the MATLAB analysis software, Ledalab V3.4.9, and processed using a continuous decomposition analysis (Benedek & Kaernbach, 2010). The continuous decomposition analysis extracted the phasic component from the tonic component of the EDA signal, providing a more accurate representation of skin conductivity for interpreting the stress reaction (Benedek & Kaernbach, 2010; Schmidt et al., 2019). The output contained the continuous phasic and tonic activity samples collected in each session, and it plotted each occurrence of challenging behavior. See Supporting Information, Items C and D, for an example of the continuous decomposition analysis plot across conditions and the phasic activity plot, respectively. See Supporting Information, Item E, for graphic examples of each step of the analysis in Ledalab V3.4.9. Features of event‐related SCRs (i.e., the frequency of significant SCRs [i.e., rapid changes in phasic conductivity at or above an amplitude threshold of 0.05 μS] within the 1–5‐s response window following the eliciting event) were extracted from the Ledalab V3.4.9 processed EDA using an open‐source MATLAB code developed by Benedek (2014), which is publicly available on the GitHub platform. These event‐related feature data were then exported into a Microsoft Excel (.xlsx) file.

The converted .csv file was then processed using convex optimization methods (Greco et al., 2015) with the open‐source software package *NeuroKit2* (Makowski et al., 2021) in Python 3 (Van Rossum & Drake, 2009) with Visual Studio Code. The convex optimization method was used to clean the EDA signal, removing noise, artifacts, and prediction and measurement errors (see Greco et al., 2015, 2016, for additional procedural details). See Supporting Information, Item F for the *NeuroKit2* processing code and Supporting Information, Items G and H for examples of the output images generated. The .csv output file contained separate continuous measures of the cleaned EDA signal samples (containing both phasic and tonic components) and the phasic and tonic components of the signal. Missing session data was excluded from both EDA extraction and processing methods (in *Ledalab* V3.4.9 and *NeuroKit2*).

Note that the negative values produced by these extraction and processing methods may indicate suboptimal readings, leading to negative values or artifacts in the analysis (Bari et al., 2024). Alternatively, these negative values could indicate a negative SCR value or a SCR potential exceeding the measurement range (Wang et al., 2024). For additional discussion of interpreting negative values, see Bari et al. (2024) and Benedek & Kaernbach (2010).

## RESULTS

### Client outcomes

**FIGURE 1:**
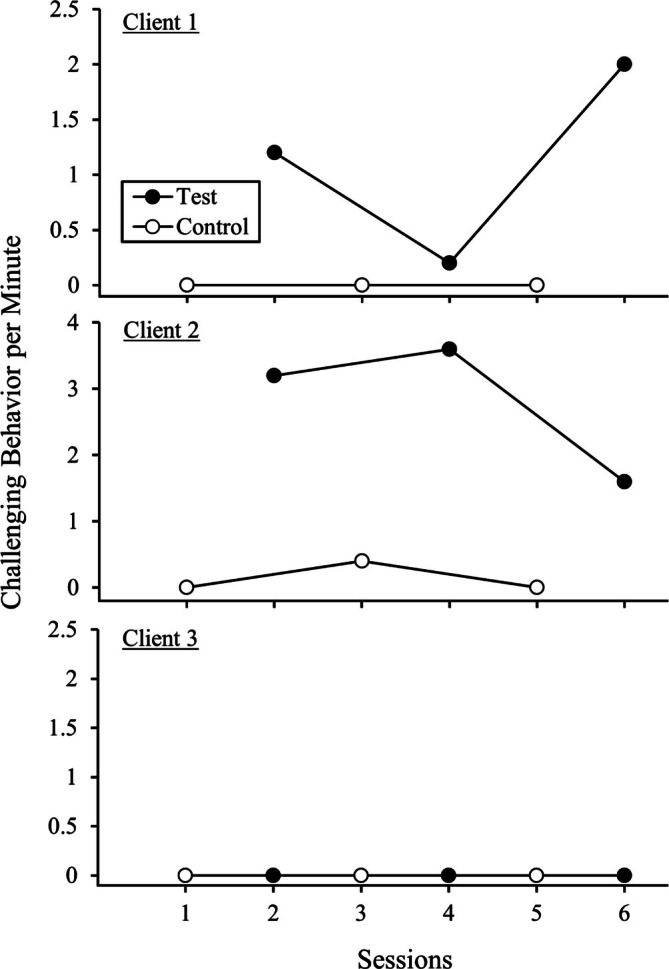
Client functional analysis results.

Figure 1 shows the client functional analysis results. For Clients 1 and 2, the functional analysis results were differentiated such that more challenging behavior occurred during test conditions and no overlap between the test and control data paths occurred. Client 3 did not exhibit any challenging behavior during the functional analysis. We retained Client 3's data in our analysis because it was an opportunity to understand how physiological measures may naturally fluctuate during a functional analysis in the absence of challenging behavior (i.e., this analysis served as an unintentional secondary source of control).

### Therapist outcomes

#### Electrodermal activity

To visualize the participant's EDA, we used violin plots to illustrate the distribution of samples across sessions. These plots were generated using the Plotly Python library (Plotly Technologies Inc., 2015). The Python scripts obtained from the library were modified using the Microsoft Copilot plug‐in in Visual Studio Code under the guidance of a clinical research associate supervised by the third author, who had skill and experience in Python programming. This process ensured compatibility with the *NeuroKit2* output and improved the visual presentation of the generated plots. See Items F–L and N–P of the Supporting Information for the code used to produce the figures, graphic depictions of data organization, and Plotly output images. To generate the figures, we used a smoothing technique (i.e., kernel density estimation), which produced a curve reflecting where the values of the EDA samples were most concentrated. Note that the shape of each violin plot may not reflect the exact minimum, maximum, or mean values due to this smoothing effect. Thus, the summary statistics provided were calculated directly from the raw data to allow for more accurate comparisons. The code provided in Items J and L of the Supporting Information produces a tool that shows these summary statistics when users hover over each plot in Visual Studio Code. For a table of the means and ranges of clean, phasic, and tonic EDA for each session across therapists, see Item M of the Supporting Information.

**FIGURE 2:**
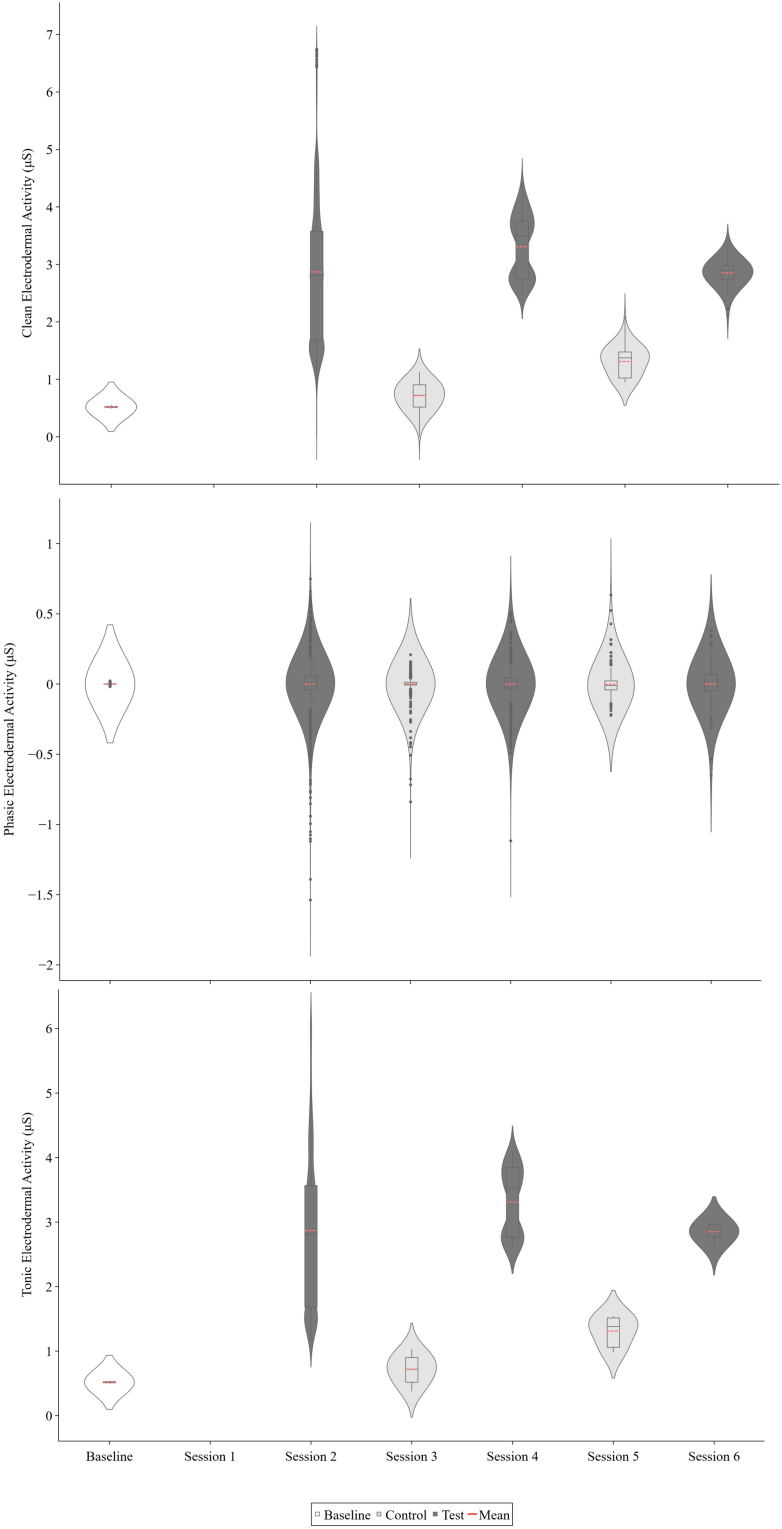
Therapist 1's clean, phasic, and tonic electrodermal activity across functional analysis sessions. The violin plot illustrates the distribution of electrodermal activity (EDA) samples collected for Therapist 1 across baseline and functional analysis sessions. The top panel shows clean EDA, the middle panel shows phasic EDA, and the bottom panel shows tonic EDA. The smoothing technique used to generate the violin plot (i.e., kernel density estimation) creates a curve reflecting where the values of the EDA samples are most concentrated. Session 1 data are not depicted due to data loss. The values on the *y*‐axis differ to best represent the reported data, as clean, phasic, and tonic EDA skew toward positive and negative values across measures.

Figure 2 displays Therapist 1's clean (top panel), phasic (middle panel), and tonic (bottom panel) EDA across functional analysis sessions. Therapist 1's clean (*M* = 3.01 μS, range: 0–6.74 μS) and tonic (*M* = 3.01 μS, range: 1.15–6.16 μS) EDA were elevated across functional analysis test sessions compared with control sessions for clean (*M* = 1.29 μS, range: 0–2.09 μS) and tonic (*M* = 1.02 μS, range: 0.38–2.99 μS) EDA and baseline for clean (*M* = 0.52 μS, range: 0.49–0.55 μS) and tonic (*M* = 0.52 μS, range: 0.50–0.54 μS) EDA. Phasic EDA showed negligible differences in differentiation across conditions. However, phasic ranges were greater during functional analysis sessions (range: −1.54–0.75 μS) than during baseline (range: −0.02–0.02 μS) and were slightly more pronounced in test sessions (range: −1.54–0.75 μS) than in control sessions (range: −0.84–0.63 μS).

**FIGURE 3:**
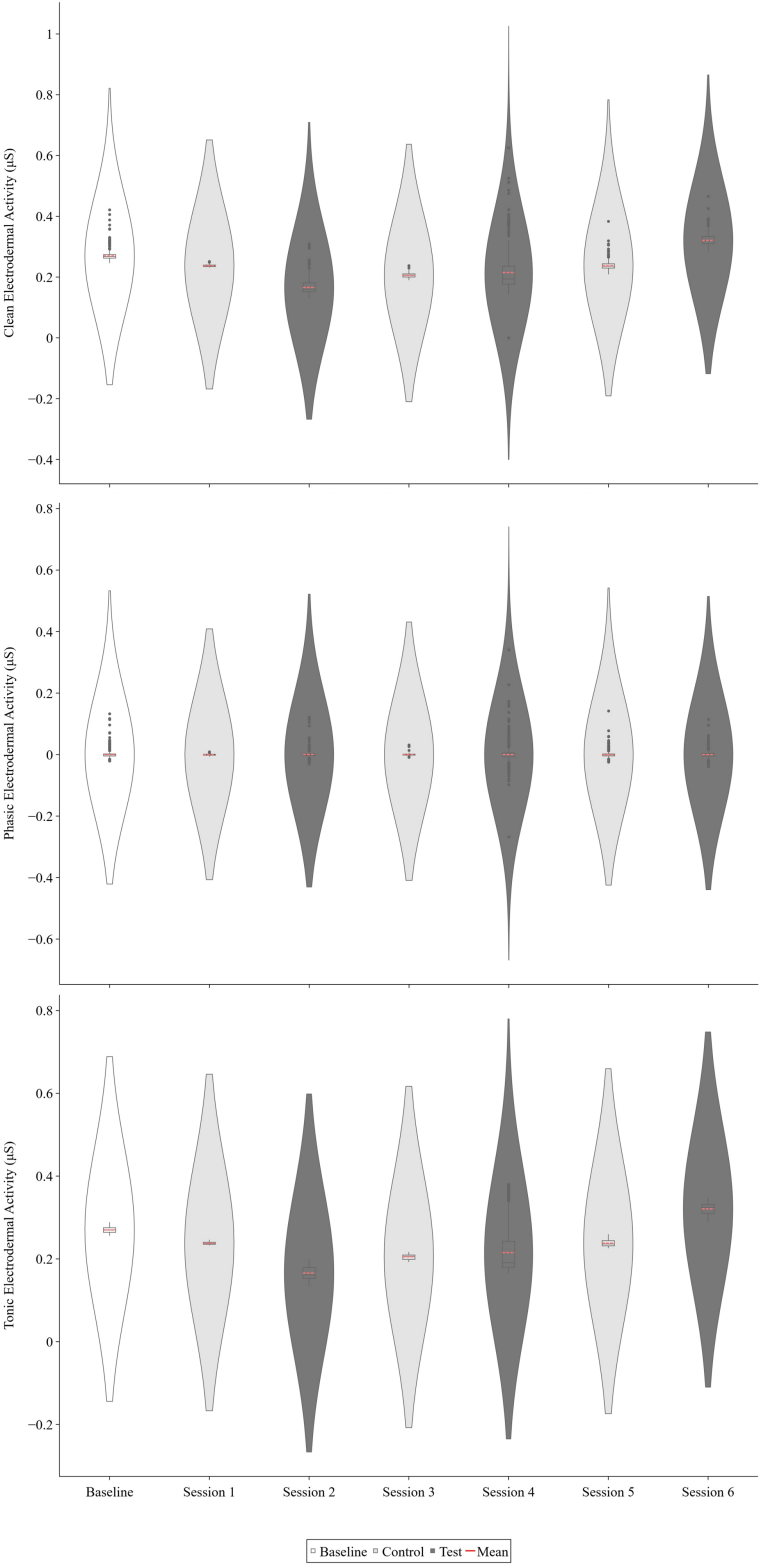
Therapist 2's clean, phasic, and tonic electrodermal activity across functional analysis sessions. The violin plot illustrates the distribution of electrodermal activity (EDA) samples collected for Therapist 2 across baseline and functional analysis sessions. The top panel shows clean EDA, the middle panel shows phasic EDA, and the bottom panel shows tonic EDA. The smoothing technique used to generate the violin plot (i.e., kernel density estimation) creates a curve reflecting where the values of the EDA samples are most concentrated. The values on the *y*‐axis differ to best represent the reported data, as clean, phasic, and tonic EDA skew toward positive and negative values across measures.

Figure 3 displays Therapist 2's clean, phasic, and tonic EDA across functional analysis sessions. Therapist 2's clean, phasic, and tonic EDA showed negligible differences in differentiation across conditions. However, overall maximum levels were slightly elevated in test sessions (0.63, 0.34, and 0.38 μS for clean, phasic, and tonic EDA, respectively) relative to the control (0.38, 0.14, and 0.26 μS for clean, phasic, and tonic EDA, respectively) and baseline sessions (0.55, 0.14, and 0.26 μS for clean, phasic, and tonic EDA, respectively).

**FIGURE 4:**
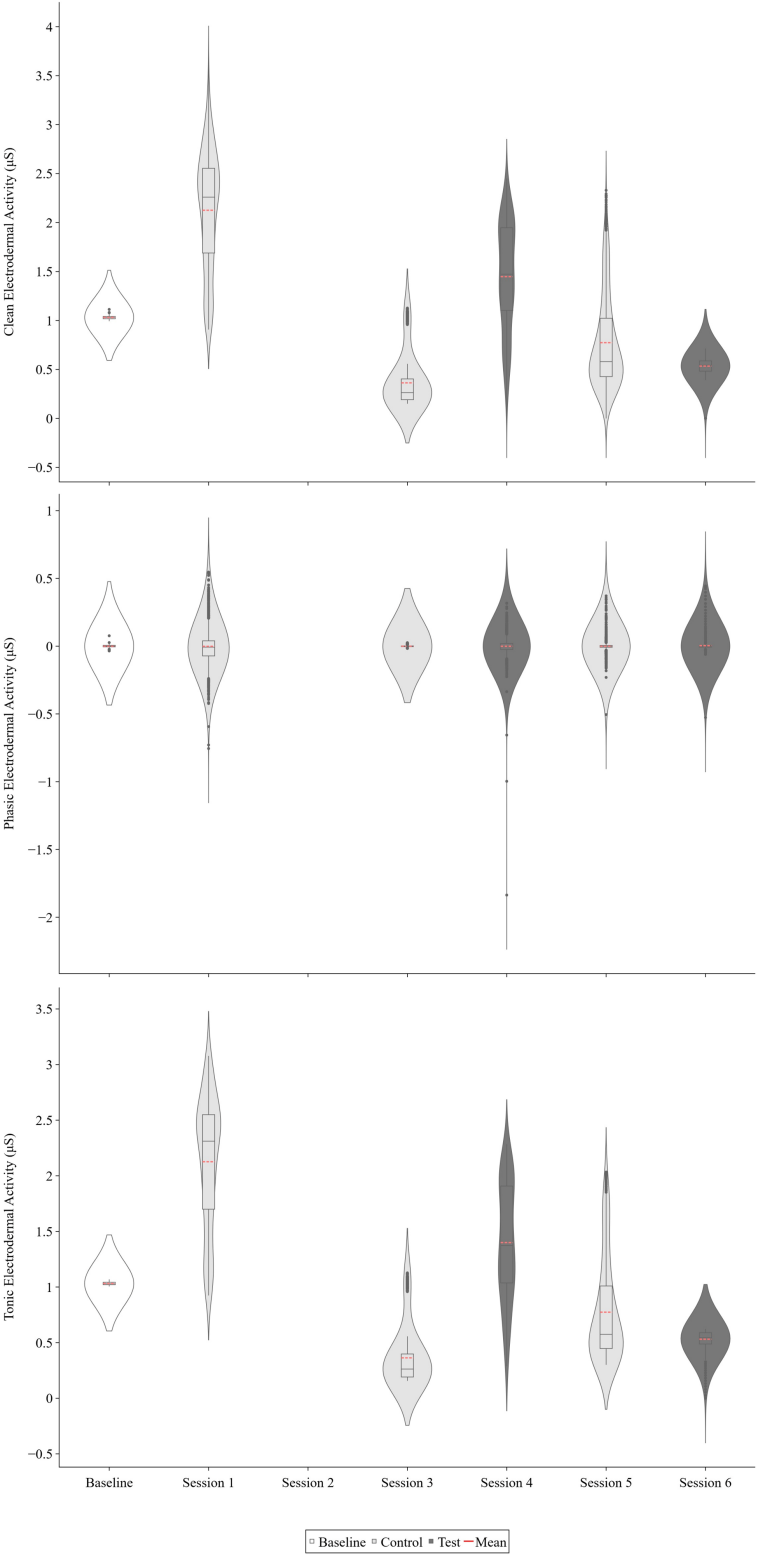
Therapist 3's clean, phasic, and tonic electrodermal activity across functional analysis sessions. The violin plot illustrates the distribution of electrodermal activity (EDA) samples collected for Therapist 3 across baseline and functional analysis sessions. The top panel shows clean EDA, the middle panel shows phasic EDA, and the bottom panel shows tonic EDA. The smoothing technique used to generate the violin plot (i.e., kernel density estimation) creates a curve reflecting where the values of the EDA samples are most concentrated. Session 2 data are not depicted due to data loss. The values on the *y*‐axis differ to best represent the reported data, as clean, phasic, and tonic EDA skew toward positive and negative values across measures.

Figure 4 displays Therapist 3's clean, phasic, and tonic EDA across functional analysis sessions. Therapist 3's clean (*M* = 2.13 μS, range: 0.91–3.61 μS) and tonic (*M* = 2.13 μS, range: 0.92–3.08 μS) EDA in Session 1 (control) were substantially elevated relative to baseline (*M* = 1.03 μS, range: 0.99–1.11 μS and *M* = 1.03 μS, range: 1–1.07 μS for clean and tonic EDA, respectively). However, across sessions, negligible differences between conditions were detected thereafter. Notably, overall levels of clean and tonic EDA appeared to decrease across the functional analysis sessions. For an aggregate depiction of therapists' clean EDA across baseline, control, and test sessions in a violin plot, see Item N of the Supporting Information.

**FIGURE 5:**
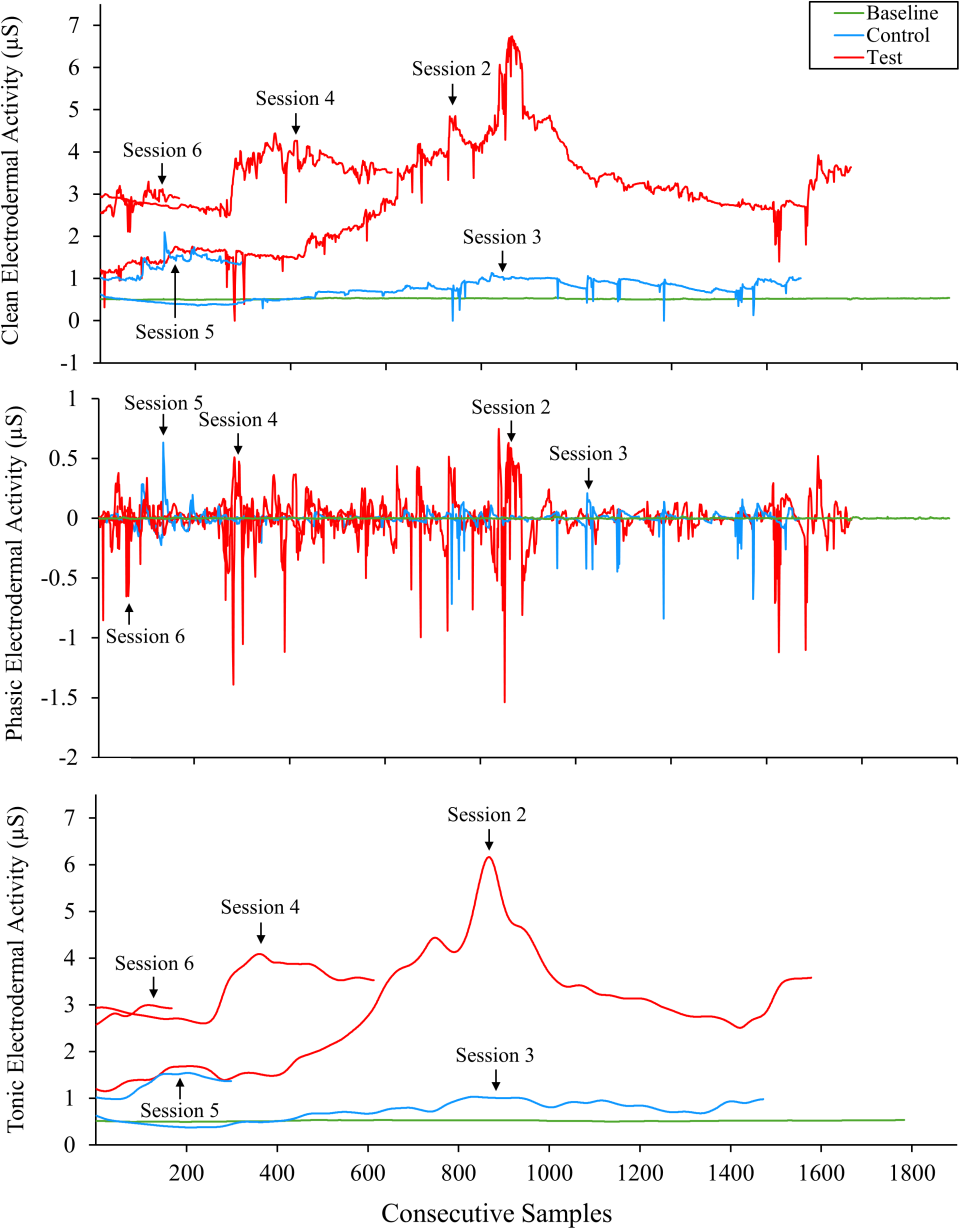
Therapist 1's electrodermal activity samples across functional analysis sessions. The electrodermal activity samples depicted are the consecutive samples collected for Therapist 1 during each functional analysis session. μS = microsiemens.

**FIGURE 6:**
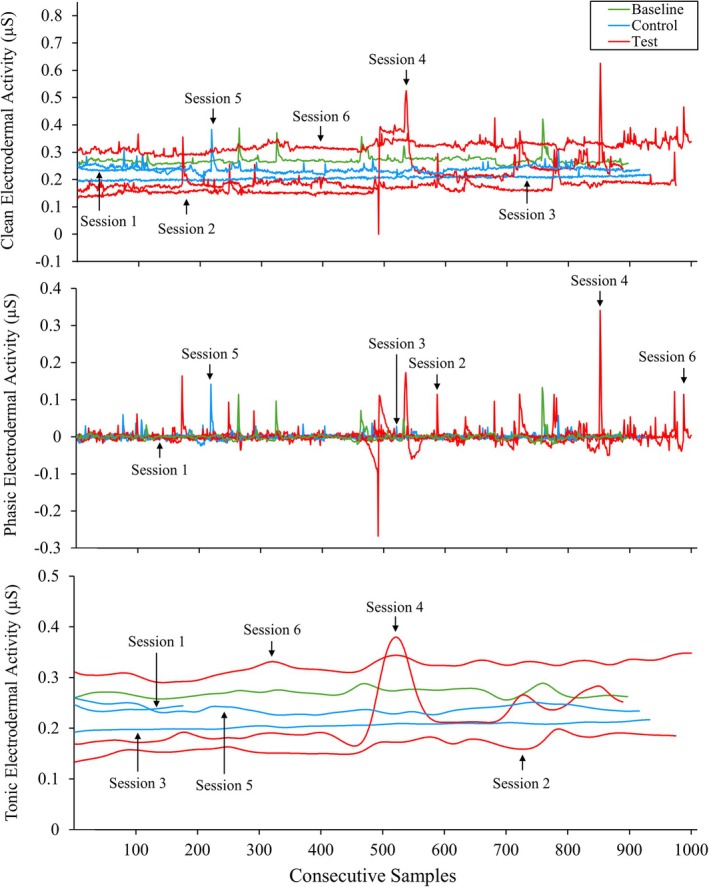
Therapist 2's electrodermal activity samples across functional analysis sessions. The electrodermal activity samples depicted are the consecutive samples collected for Therapist 2 during each functional analysis session. μS = microsiemens.

**FIGURE 7:**
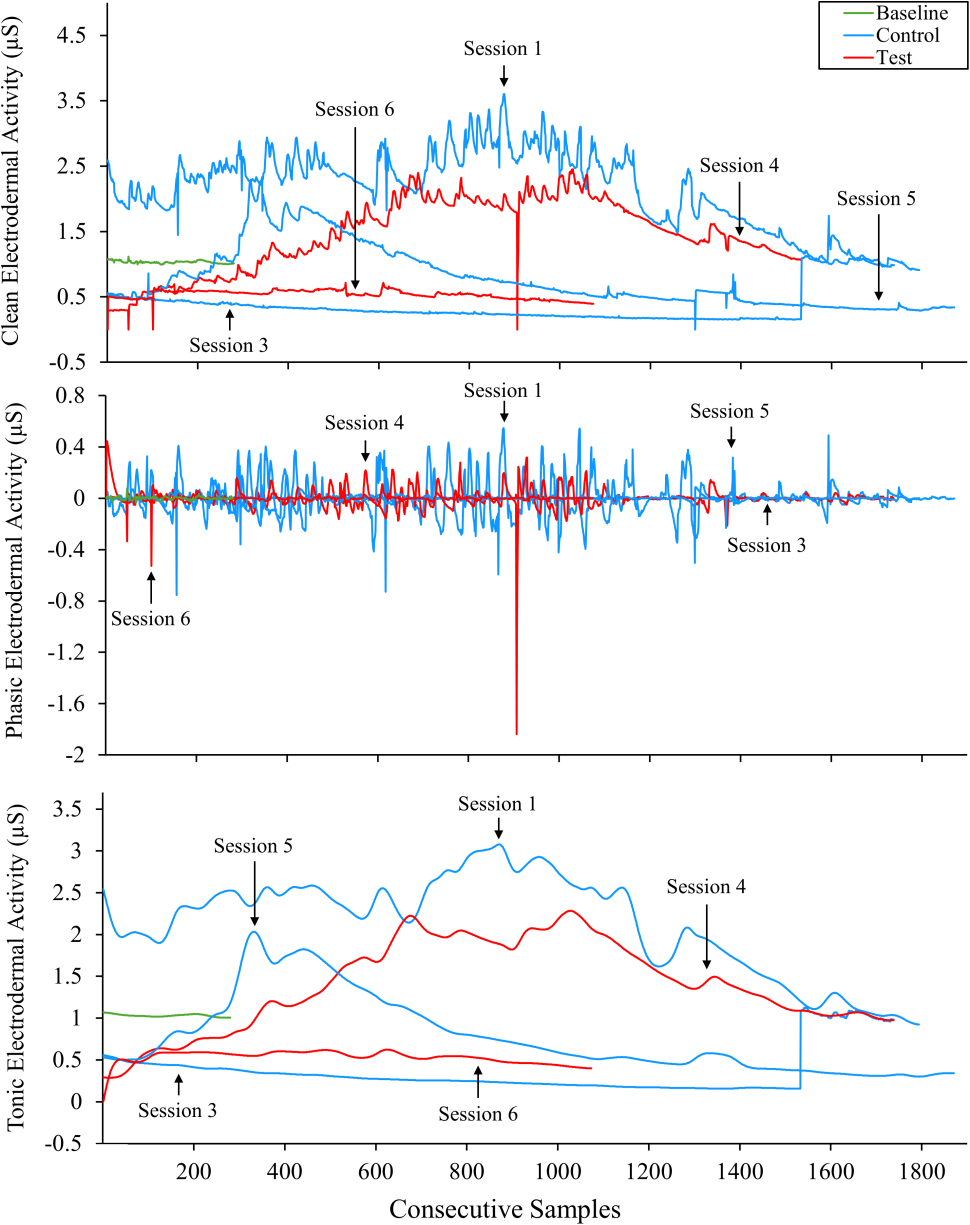
Therapist 3's electrodermal activity samples across functional analysis sessions. The electrodermal activity samples depicted are the consecutive samples collected for Therapist 3 during each functional analysis session. μS = microsiemens.

To show within‐session changes in therapist clean, phasic, and tonic EDA, we used line graphs to display the consecutive samples collected in baseline and functional analysis sessions. Figures 5, 6, and 7 show EDA samples across the functional analysis sessions for Therapists 1, 2, and 3, respectively. Therapist 1's clean and tonic EDA (Figure 5) show higher levels in test sessions than in control and baseline sessions. Test session samples appear to increase across the session. In one test session with the greatest total samples collected (Session 2), clean and tonic EDA trend upward until peak activity is observed, then levels slowly descend and begin to decrease again toward the end of the session. Phasic EDA shows greater variability (fast level changes) in functional analysis sessions than during baseline and to a greater extent in test sessions relative to control sessions. Session 1 (control) data for Therapist 1 were lost, which may be one reason for these patterns. Therapist 2's clean and tonic EDA (Figure 6) shows greater variability across functional analysis sessions relative to baseline, with minimal differences between test and control sessions, except for a single test session (Session 6). In Session 6, overall clean and tonic EDA levels were elevated relative to the other functional analysis sessions and baseline. Greater variability in phasic EDA is observed across consecutive samples in functional analysis sessions relative to baseline, and this variability is more pronounced in test sessions (see Figure 7, Therapist 2 clean EDA in Test Sessions 2, 4, and 6 [top panel] and tonic EDA in Test Session 4 [bottom panel]). Therapist 3's clean and tonic EDA (Figure 7) generally appear elevated in control sessions, at least at the start (see Control Sessions 1 and 5), relative to test sessions (see Test Sessions 4 and 6; the exception is Control Session 3, which shows decreased levels relative to test sessions). No challenging behavior occurred during Therapist 3's functional analysis, which may have contributed to inconsistent patterns across test and control sessions and relative to the results of Therapists 1 and 2. Phasic EDA shows overall greater variability in control sessions; however, this may reflect the comparatively fewer samples collected during test sessions, as Test Session 2 data were lost.

#### Skin conductance responses

**FIGURE 8:**
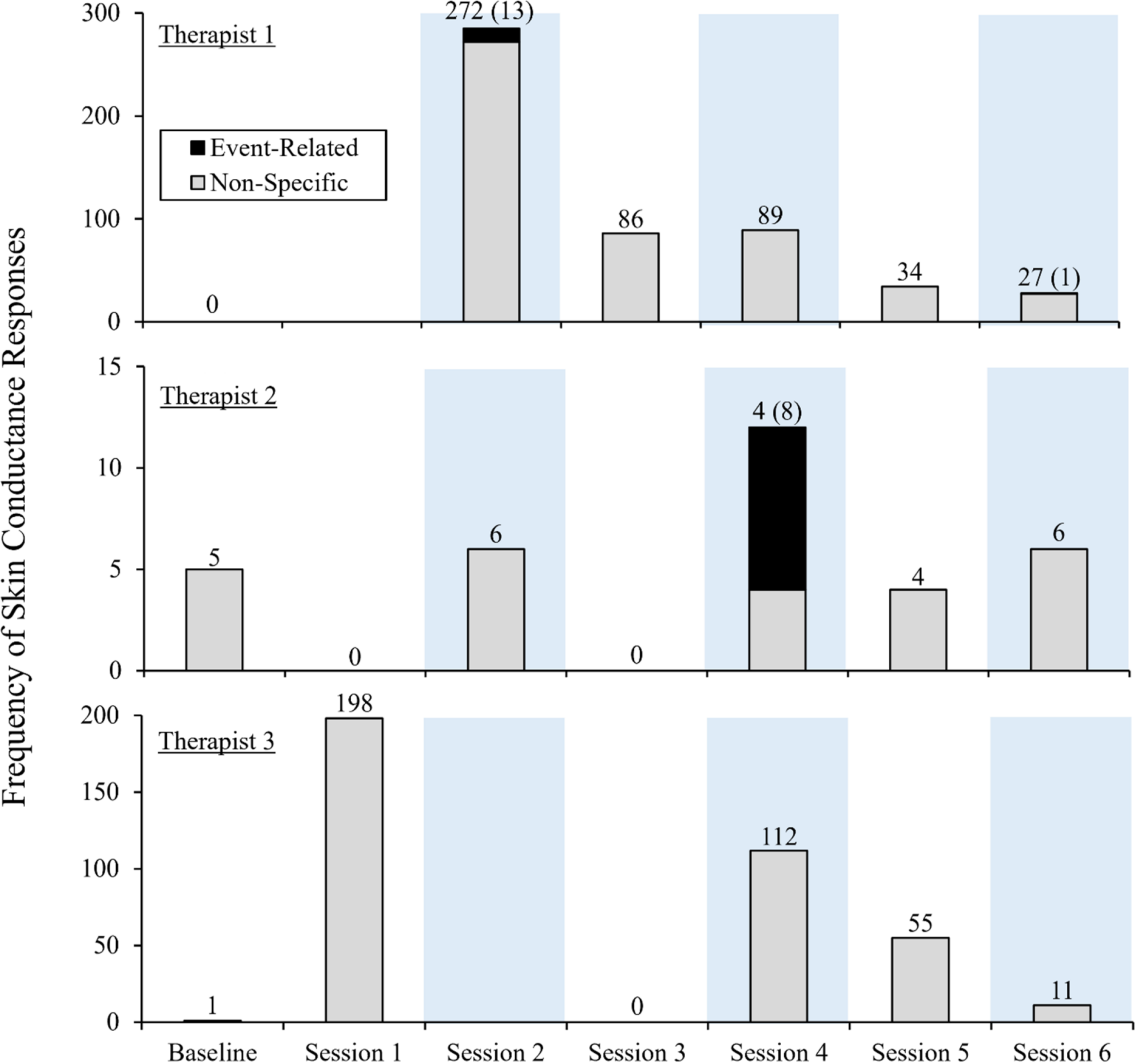
Therapist skin conductance responses across functional analysis sessions. The light blue shading depicts test sessions. The data labels show the frequency of nonspecific skin conductance responses, and the numbers in parentheses are the frequency of event‐related responses.

Figure 8 depicts the frequency of therapists' nonspecific and event‐related SCRs across baseline and functional analysis sessions. For Therapist 1, Test Sessions 2 (13 event‐related, 272 nonspecific), 4 (zero event‐related, 89 nonspecific), and 6 (one event‐related, 27 nonspecific) produced a substantially lower proportion of event‐related SCRs (4.56, 0, and 3.57%, respectively) than nonspecific SCRs (95.44, 100, and 96.43%). Test Session 4 was the only session during which Therapist 2 exhibited event‐related SCRs (eight event‐related, four nonspecific). Unlike Therapist 1, Therapist 2's event‐related SCRs were proportionally greater (66.67%) than nonspecific SCRs (33.33%) in the single session during which they occurred. Therapist 3 exhibited no event‐related SCRs, as challenging behavior did not occur during their analysis. However, Therapist 3 exhibited nonspecific SCRs in baseline (one) and Sessions 1 (198), 4 (112), 5 (55), and 6 (11). A greater proportion of nonspecific SCRs occurred in control sessions (67.29%) than in test sessions (32.71%). However, this may be because these values reflect calculations from three control sessions and only two test sessions (data from Test Session 2 were lost). Overall, the frequency of nonspecific SCRs appeared to decrease across functional analysis sessions.

Table 1 displays the challenging behavior topographies exhibited by Clients 1 and 2 that produced each event‐related SCR and the mean skin conductance value (mean clean EDA values within the 5‐s period following the corresponding occurrence of challenging behavior) for Therapists 1 and 2. Note that the values reported in Table 1 include only sessions and instances of challenging behavior for which EDA samples were recorded; missing data were excluded (see Data loss below). The challenging behavior topographies that elicited event‐related SCRs included hitting, grabbing, and pinching for Therapist 1 and throwing an item and hand‐to‐head self‐injury for Therapist 2. A hit that contacted Therapist 1's neck elicited the greatest number of event‐related SCRs in the 5‐s period following the response (three event‐related SCRs). An occurrence of hand‐to‐head self‐injury that did not contact Therapist 2 elicited the greatest number of event‐related SCRs following the response (two event‐related SCRs).

**TABLE 1:** Therapists 1 and 2 event‐related skin conductance response details.

	Challenging behavior topography	Site of therapist contact	Event‐related skin conductance responses	Mean skin conductance value (μS)
Therapist 1				
Session 2 (Test)				
	Hit	Lower right arm	2	1.25
	Hit	Right hand and abdomen	1	1.59
	Grab/Pinch	Posterior thighs	2	2.39
	Hit	Neck	2	**3.43**
	Grab	Neck	2	**3.49**
	Hit	Right hip	2	**4.48**
	Hit	Right hip	2	**4.55**
	Grab	Neck	1	**4.64**
	Hit	Neck	3	**5.57**
	Hit	Left hand	2	2.72
	Hit	Left hand	1	**2.99**
	Hit	Left hand	2	**3.24**
	Hit	Left hand	2	**3.33**
Session 6 (Test)				
	Hit	Right hip	1	2.69
Therapist 2				
Session 4 (Test)				
	Throw Item	Right hand	1	**0.42**
	Hand‐to‐head self‐injury	‐	1	**0.43**
	Hand‐to‐head self‐injury	‐	1	**0.42**
	Hand‐to‐head self‐injury	‐	1	**0.24**
	Hand‐to‐head self‐injury	‐	2	**0.25**
	Hand‐to‐head self‐injury	Left hand	1	**0.28**
	Hand‐to‐head self‐injury	Right hand	1	**0.30**

*Note*: The total event‐related skin conductance responses and the mean skin conductance value reflect the 5‐s period following the corresponding occurrence of challenging behavior. Bold mean skin conductance values depict those above the mean values for therapist clean electrodermal activity (EDA) for each session (2.87 μS and 2.85 μS for Therapist 1, Sessions 2 and 6, respectively; 0.21 μS for Therapist 2, Session 4). The values reported include only sessions and instances of challenging behavior for which EDA samples for Therapists 1 and 2 were recorded; missing EDA samples were excluded.

Therapist 1 experienced nine instances of challenging behavior (all occurred in Test Session 2), which produced an event‐related SCR, with a mean skin conductance value greater than the mean clean EDA value across the session (*M* = 2.87 μS, range: 1.25–5.57 μS). A total of four instances of challenging behavior did not produce a mean skin conductance value greater than the mean clean EDA value across the session (three in Session 2 and one in Session 6). Therapist 2 experienced seven instances of challenging behavior in Test Session 4, which produced a mean skin conductance value greater than the mean clean EDA value across the session (*M* = 0.21 μS, range: 0.24–0.43 μS).

The conditional probability of an event‐related SCR given challenging behavior and the unconditional probability of an SCR (nonspecific or event‐related) occurring at any point across all sessions were evaluated for Therapists 1 and 2 (data not depicted). For Therapist 1, 14 event‐related SCRs and 34 instances of challenging behavior occurred during the functional analysis. The conditional probability of an event‐related SCR following challenging behavior was 0.41 (14 event‐related SCRs divided by 34 instances of challenging behavior), and the unconditional probability of an SCR was 0.18 (522 total SCRs divided by 2,827.2 s of total session time with lost data excluded). For Therapist 2, there were a total of eight event‐related SCRs and 44 instances of challenging behavior during the functional analysis. For Therapist 2, the conditional probability of an event‐related SCR following challenging behavior was 0.18 (eight event‐related SCRs divided by 44 instances of challenging behavior), and the unconditional probability of an SCR was 0.02 (33 total SCRs divided by 1,877.4 s of total session time with lost data excluded).

#### Data loss

Missing EDA samples were excluded from the data analysis. These samples were likely lost or missing during the data extraction process due to electrode misalignment with the skin or data transmission errors (Böttcher et al., 2022). Overall, data loss accounted for 30.41% of total session time across the three therapists (53.22 min of 175 min total). No data from baseline sessions were lost. Control session data loss included 34.31% of total session time (25.73 min of 75 min total), and test session data loss included 36.64% of total session time (27.48 min of 75 min total). Data loss included 44.29% (31 min, including all of Session 1) of session time for Therapist 1, 11.86% (4.15 min of 35 total min) for Therapist 2, and 25.79% (18.05 min of 75 total min, including all of Session 2) for Therapist 3. This level of data loss falls within the 10% to 50% range reported in wearable technology studies evaluating the quality of data recorded using the Empatica E4 (Böttcher et al., 2022). Although no wearable wristband currently has the capability of measuring EDA with complete accuracy in ambulatory or naturalistic settings (Milstein & Gordon, 2020), processing these data samples using artifact recognition, as was done in this study, may improve precision and overall quality (Böttcher et al., 2022).

## DISCUSSION

This technical report demonstrates the use of wearable technology to measure therapist EDA during assessments of challenging behavior. Whereas prior research has leveraged wearable technology to monitor clients' physiological responses in behavioral assessment (e.g., Ellement et al., 2021; McCabe & Greer, 2023; Zheng et al., 2021), the application of this technology to evaluate therapists' physiological responding was a novel feature of the current study. Working with clients with challenging behavior comes with inherent risks. Although numerous tactics have been used to mitigate physical injury, obtaining supplemental measures of physiological responding can help inform additional efforts to support therapist safety and well‐being.

Our preliminary results with three therapist–client dyads revealed idiosyncrasies that should be anticipated when measuring physiological arousal. For one participant (Therapist 1), EDA and SCR measures indicated heightened physiological arousal during functional analysis sessions relative to baseline (sitting in a chair), with consistently higher mean and maximum EDA and SCR values during test sessions than during control sessions. Therapist 2 showed comparatively less differentiation in measures across baseline, test, and control sessions. However, summaries of maximum EDA and SCR frequency were higher in functional analysis test sessions than in control sessions and baseline. Additionally, for both therapists, the conditional probability of an SCR given challenging behavior was higher than the unconditional probability of an SCR occurring at any point during sessions.

Behavior‐analytic experience (Therapist 1 had substantially more experience in the setting but less overall behavior‐analytic training), amount of challenging behavior experienced (Therapist 1's event‐related SCRs were related to aggression, whereas Therapist 2's were largely related to client self‐injury), or both may be partial explanations for the differences in EDA and SCR measures between Therapist 1 and 2. Additional reasons for these differences may be related to data loss (Therapist 2 had the most complete data set) and other uncontrolled factors in the current study. Therapist 3's results deviated from those of Therapists 1 and 2, as physiological arousal seemed to peak during implementation of the first control session and decreased across subsequent functional analysis sessions. For this therapist, fewer SCRs and lower EDA elevation occurred in test sessions than in control sessions. It is possible that Therapist 3's response pattern differed because challenging behavior did not occur. That is, Therapist 3's data set may reflect differences in physiological experiences related to anticipating challenging behavior versus experiencing its occurrence. Previous research has shown that skin conductivity can increase in magnitude during periods of anticipation and may exceed levels during the precipitating event (i.e., stressor; Nomikos et al., 1968). Overall decreased EDA levels across functional analysis sessions might indicate that after an initial anticipatory period, Therapist 3 either perceived a decreased likelihood of challenging behavior or habituated to implementing the functional analysis conditions. Emerging research has begun applying machine‐learning algorithms to biomarkers of stress (including EDA) to distinguish anticipatory and acute stress reactions (Andrić et al., 2024). Applying similar approaches in future investigations may provide greater clarity regarding divergent findings in settings in which exposure to stressors is less controlled.

It is also important to consider features of the functional analysis other than challenging behavior that may have influenced changes in therapist EDA. For one, the therapist's movement and physical effort during the functional analysis were neither controlled nor measured. This included arranging antecedent conditions (e.g., play in the control condition or delivering instructions in the test condition) and consequence conditions (e.g., attention and tangible delivery) associated with each session type. Previous researchers have suggested incorporating movement measures (e.g., accelerometry) in conjunction with EDA to improve data quality (Böttcher et al., 2022) and the robustness of stress detection (Betti et al., 2018). Including these measures may improve the precision with which the results of future investigations can be interpreted. In addition, client behavior was not controlled in this study. Thus, unmeasured behavior may have fluctuated across sessions, including nontargeted challenging behavior, cooperation, and general movement around the room. Future research exploring EDA changes in relation to the occurrence of challenging behavior may benefit from recording more events—for example, recording nontarget client responding, caregiver or peer responses, or implementation of de‐escalation or physical crisis management strategies. Moreover, future research may more tightly control for extraneous variables by using confederate clients who exhibit programmed responses across relevant settings and situations in which challenging behavior typically occurs.

Although this study provides preliminary evidence of the feasibility of using wearable technology to monitor therapists' EDA during the assessment of challenging behavior, several limitations should inform future research. Foremost, this study was conducted in a university outpatient clinic that had the necessary resources to collect these physiological measures and process and analyze the data. We recognize that these resources are not accessible in all behavior‐analytic service delivery settings. Therefore, we offer several potential reasons why incorporating physiological measures in this study was feasible. First, our research team included interdisciplinary professionals, specifically one with experience using these measures in previous research (third author), who was involved in the design and execution of the procedures. Second, the software program cometrics was developed at the clinic's affiliated university, and our research team's familiarity with the program likely facilitated ease of use and supported the development of compatible data processing and analysis procedures (i.e., Python 3 code in MATLAB, *NeuroKit2*, and Plotly). See Items B–L and O–R of the Supporting Information for the resources developed for data processing and analysis in the current study. Third, the therapists participating in this study were employed by a clinic in which conducting research was a primary focus, which may have influenced their willingness to participate. In nonresearch settings, therapists may be less likely to consent to wearing devices that measure and share health data with others. Given the benefits of wearable technology for monitoring stress responses, researchers should consider developing safeguards and best‐practice guidelines to address potential ethical concerns related to physiological monitoring in both research and practice.

Although the measures included in this study provide valuable insights into how physiological arousal increases when encountering challenging behavior, future applications should be complemented by the use of empirically validated self‐report measures to better contextualize how individuals experience these changes (Crosswell & Lockwood, 2020). Notably, our results had no bearing on whether the therapists perceived physiological changes as adaptive or maladaptive. Research has shown that moderators of nonadaptive outcomes include negative emotional reactions and fear of experiencing challenging behavior (Rose et al., 2013). Thus, precision of physiological stress measures combined with the use of valid and reliable self‐report measures may improve how the various responses individuals exhibit when exposed to common stressors are interpreted (Christopoulos et al., 2019).

Thus, we recommend that the physiological experiences of all who are regularly exposed to challenging behavior be given equal consideration in future research. Exploring the physiological experiences of those who facilitate assessments and interventions to address challenging behavior may yield greater insight into factors influencing not only the development of challenging behavior but also fidelity, adherence, and effectiveness (Allen & Warzak, 2000; Stocco & Thompson, 2015).

### Technological considerations

A well‐documented limitation of wearable technology is data loss and low data quality. Data loss in this study fell within the 10% to 50% range (i.e., 38.14%) typically reported in wearable technology studies evaluating the Empatica E4 (Böttcher et al., 2022). The data loss and abnormality issues may be related to malfunctions in the recording process itself (Tronstad et al., 2022). Researchers using wearable technology in ambulatory settings have also reported that a loose connection between the electrodes and the skin is a major source of data loss (Coffman et al., 2020). A proposed strategy to mitigate these concerns is to ensure real‐time monitoring of the EDA signal so that if all recorded values are approximately 0 (an indicator of a loose connection), timely adjustments can be made to more tightly secure the technology (Coffman et al., 2020). Although real‐time monitoring of the EDA signal was employed in this study using *cometrics*, we did not systematically use these data to monitor the appropriate electrode–skin connection. We recommend real‐time monitoring and adjustment, when possible. Additionally, some data loss may be attributed to reliance on Bluetooth connections, which can be interrupted by physical barriers, such as the one‐way glass in clinic therapy rooms. Moving forward, adopting open‐source hardware options, such as the EmotiBit wearable sensor (Montgomery et al., 2023), which transmits data via more robust wireless internet connections, could mitigate data loss and enhance research accessibility.

Various sources of artifacts in the recorded signal also pose a risk to data quality and can be produced by the therapists' physiological activity (e.g., deep inhalations, changes in respiratory patterns) and the electrode–skin connection (e.g., detachment; Tronstad et al., 2022). Although artifacts compromising accuracy are common when measuring EDA in naturalistic or ambulatory settings (Milstein & Gordon, 2020), researchers suggest that processing these data using artifact recognition may improve the overall data quality (Böttcher et al., 2022). In this study, EDA data were processed using continuous decomposition analysis (Benedek & Kaernbach, 2010) and convex optimization methods (Greco et al., 2015). Both methods employ robust data processing, including artifact recognition and correction, producing clean EDA signals and feature outputs that are likely to improve data quality.

In the current study, real‐time output was generated using *cometrics*, but analysis of the therapists' EDA was completed post hoc. One exciting extension to this work, therefore, is to evaluate the feasibility of allowing therapists to monitor their own physiology in real time, perhaps signaling when procedural adjustments—such as switching therapists during a functional analysis—are needed based on the output. A recent client‐focused study by Emezie et al. (2024) highlighted the evolving capabilities of wearable technology in enabling real‐time monitoring. Emezie et al. examined the efficacy of the KeepCalm smartphone application, a digital mental health interface that monitors clients' physiological responding in real time. Based on biomarkers of stress (i.e., heart rate), the application suggests in‐the‐moment intervention strategies to caregivers to address client stress and co‐occurring challenging behavior. The study demonstrated that use of the intervention strategies suggested by the KeepCalm application led to reductions in client heart rate. Moreover, an extension by Kaur et al. (2025) suggested that stakeholders found the application easy to use. However, these authors noted that further evaluation of the KeepCalm application is needed at the implementation and maintenance phases. With such promising innovations focused on client outcomes, it is also worth considering how wearable technology can advance therapist outcomes.

Of course, practical and cost‐effective solutions are critical for widespread adoption. Open‐source platforms for wearable technology provide accessible entry points for practitioners to begin monitoring and addressing therapist stress. The Empatica E4 device used in this study is no longer commercially available. Although its next‐generation counterparts record similar metrics, they are nearly three times more expensive than open‐source alternatives. The updated proprietary devices are available for purchase; however, they impose mandatory subscription fees and necessitate third‐party data processing. Therefore, we encourage future researchers to consider using devices like the EmotiBit, which, like the Empatica E4, are designed to capture a wide range of physiological and movement data. The EmotiBit is well‐suited for research applications that require detailed insights into human physiology and emotional responses, providing similar measures and data quality (Montgomery et al., 2023, 2024). Importantly, these devices are available at a fraction of the cost of the leading physiological recording devices (Montgomery et al., 2023). However, these open‐source options may require some level of technical expertise. For example, using EmotiBit in combination with *cometrics* would require the additional development of both open‐source projects. Additionally, coding expertise in MATLAB and Python 3 is likely also required to properly process EDA data using packages such as *Ledalab* V3.4.9 and *NeuroKit2*. Future studies should assess the accessibility and feasibility of less expensive open‐source devices for evaluating therapist stress.

Finally, suggested best practices for EDA measurement vary across published research (Horvers et al., 2021). Due to the unique designs of studies in which EDA is measured, gold‐standard recording and processing procedures do not exist. This lack of standards presents a risk of researchers misinterpreting the metric (Li et al., 2022). Thus, we encourage future research to involve collaborative, interdisciplinary research teams, such as the one in our study. Additional collaborators to consider include physiologists, neuroscientists, software developers, and related professionals with expertise in collecting, analyzing, and interpreting physiological data. The interdisciplinary team that collaborated on the development and execution of this study included two doctoral‐level board‐certified behavior analysts specializing in the assessment and treatment of challenging behavior and a doctoral‐level data scientist with expertise in neuroscience, engineering, and virtual reality software development. Although this improved our ability to process and analyze therapist EDA data, substantial effort was put forth to familiarize ourselves with relevant research on EDA, physiological stress, and wearable technology, topics that remain scarce in behavior‐analytic journals. We hope that the current technical report facilitates future interdisciplinary collaborations by reducing the time and effort required to access, interpret, and implement EDA measures in behavior‐analytic research.

## AUTHOR CONTRIBUTIONS

Emily K. Sullivan conceptualized the research study and designed the methodology, conducted the experiment, analyzed and processed the data, created data visualizations, prepared the original manuscript, and revised subsequent drafts of the manuscript. Tara A. Fahmie contributed to the methodology, data visualizations, writing the original manuscript, and revising subsequent drafts. Jamie E. Gehringer contributed to the conceptualization of the research study, data analysis and processing, data visualizations, writing the original manuscript, and revising subsequent drafts.

## CONFLICT OF INTEREST STATEMENT

The authors declare no conflicts of interest.

## ETHICS APPROVAL

This study received institutional review board approval and was conducted in accordance with established ethical guidelines for the treatment of human participants.

## Supporting information


**Data S1** Supporting Information

## Data Availability

The data that support the findings of this study are available from the corresponding author on request. Supporting Information A includes a depiction of the cometrics Platform. Supporting Information files B, F, H, J, and L include all code used in the current study for data processing and visualization. Supporting Information files C, D, and E include examples of the Ledalab V3.4.9 Continuous Decomposition Analysis across conditions, phasic activity across conditions, and a graphic example, respectively. Supporting Information G includes an example of the NeuroKit2 processing output images. Supporting Information I includes an example of the organization of the (.xlsx) file for the NeuroKit2‐processed data. Supporting Information K includes an example of the output image for the condition‐aggregate Plotly violin plot. Supporting Information M includes a table of the therapists' mean clean, phasic, and tonic electrodermal activity across functional analysis sessions. Supporting Information files N–R include various examples of how the electrodermal activity data of the Therapists in this study could be visualized using violin plots created in Plotly. All Supporting Information files can also be accessed using the Open Science Framework Data Repository referenced and linked in the Supporting Information file.

## Footnotes

^1^
The participants of this study also participated in a study reported in a manuscript published in the *Journal of Organizational Behavior Management* on May 5, 2025 (https://doi.org/10.1080/01608061.2025.2504947). The purpose of this study, data reported, and the figures included are distinct from those reported in the manuscript published in the *Journal of Organizational Behavior Management*.

^2^
The Destructive Behavior Severity Scale was used to document the severity of injury risk posed by challenging behavior; however, the reliability and validity of the scale have yet to be formally evaluated.
