# Supplementary material for: Using wearable technology to evaluate the electrodermal activity of therapists assessing challenging behavior
Source: J Appl Behav Anal. 2026 Jan 23;59(1):e70050. doi: 10.1002/jaba.70050 (PMC12828444; doi:10.1002/jaba.70050)

**Supporting Information Table of Contents**

| **Item** | **Title/Description** |
| --- | --- |
| A | The cometrics Platform |
| **B** | **MATLAB E4 Output File (.json) to Excel File (.xlsx) Code** |
| C | Example of Ledalab V3.4.9 Continuous Decomposition Analysis Across Conditions |
| D | Example of Ledalab V3.4.9 Continuous Decomposition Analysis Phasic Activity Across Conditions |
| E | Therapist 1 Test Session 2 Graphic Example of LedaLab Continuous Decomposition Analysis |
| **F** | **NeuroKit2 Electrodermal Activity Output (.csv) Processing Code** |
| G | Therapist 2 Test Session 2 Example NeuroKit2 Processing Output Images |
| **H** | **Therapist 2 Test Session 2 Example NeuroKit2 Processing Output Images (additional event examples)** |
| I | Example of (.xlsx) File Organization of Neurokit2 Processed Data (.csv) for Ploty Graphing |
| **J** | **Condition Aggregate Plotly Violin Plot from Neurokit2 Processed File (.csv) Code** |
| K | Therapist 1 Example Output Image of Condition Aggregate Plotly Violin Plot |
| **L** | **Separate Conditions Plotly Violin Plot from Neurokit2 Processed File (.csv) Code** |
| M | Therapist Mean Clean, Phasic, and Tonic Electrodermal Activity Across Functional Analysis Sessions |
| N | Therapist Clean Electrodermal Activity Across Functional Analysis Conditions |
| O | Therapist 1 Example Output Image of Separate Conditions Plotly Violin Plot |
| P | Therapist 1 Clean, Phasic, and Tonic Electrodermal Activity Across Conditions |
| Q | Therapist 2 Clean, Phasic, and Tonic Electrodermal Activity Across Conditions |
| R | Therapist 3 Clean, Phasic, and Tonic Electrodermal Activity Across Conditions |

*Note.* Bolded items can be found in the associated Open Science Framework (OSF) Data Repository. Access link: <https://osf.io/v98yb/?view_only=ee7b0d359f8a46388f671c0fd3ec9124>

**Item A**

*The cometrics Platform*

*
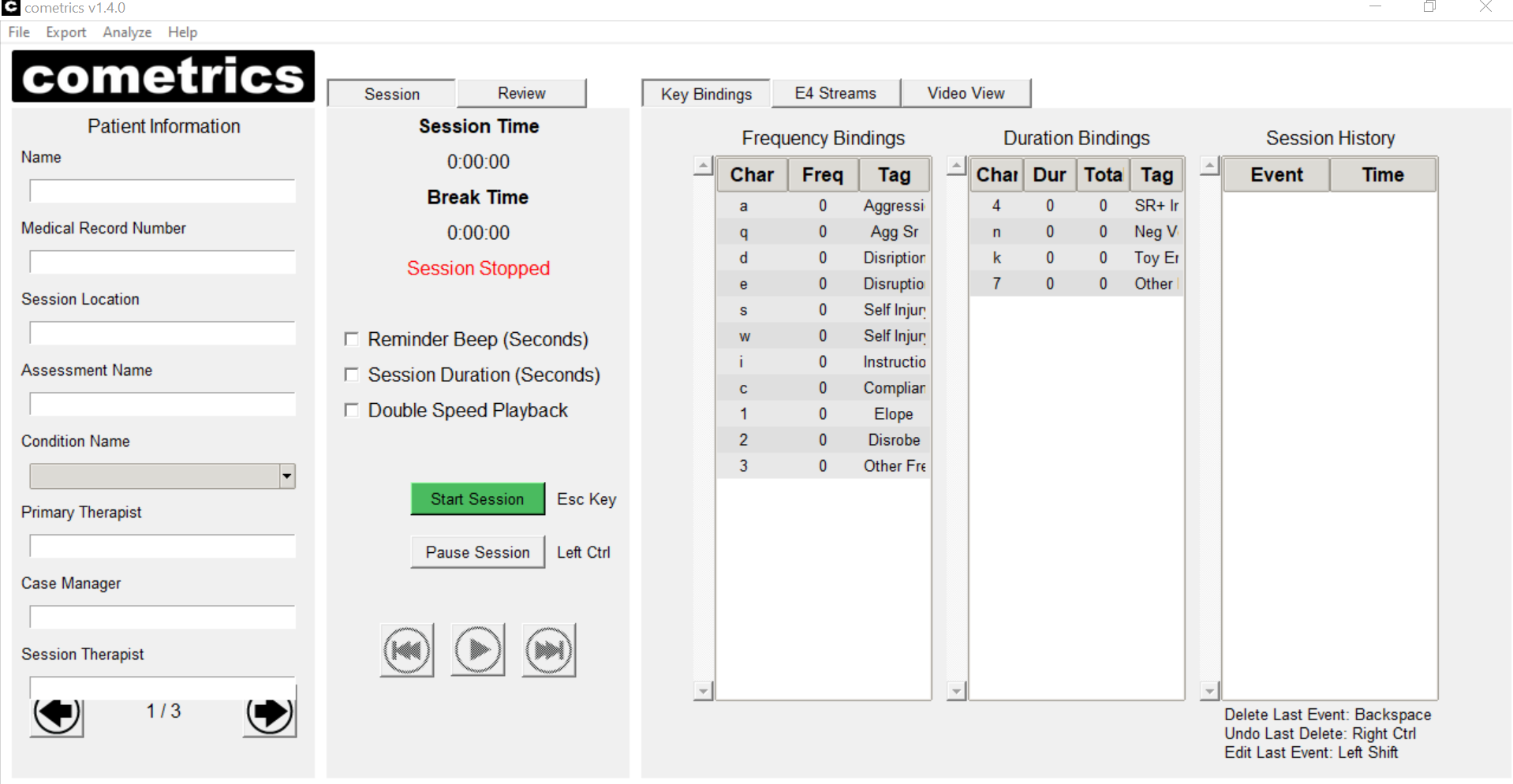
*


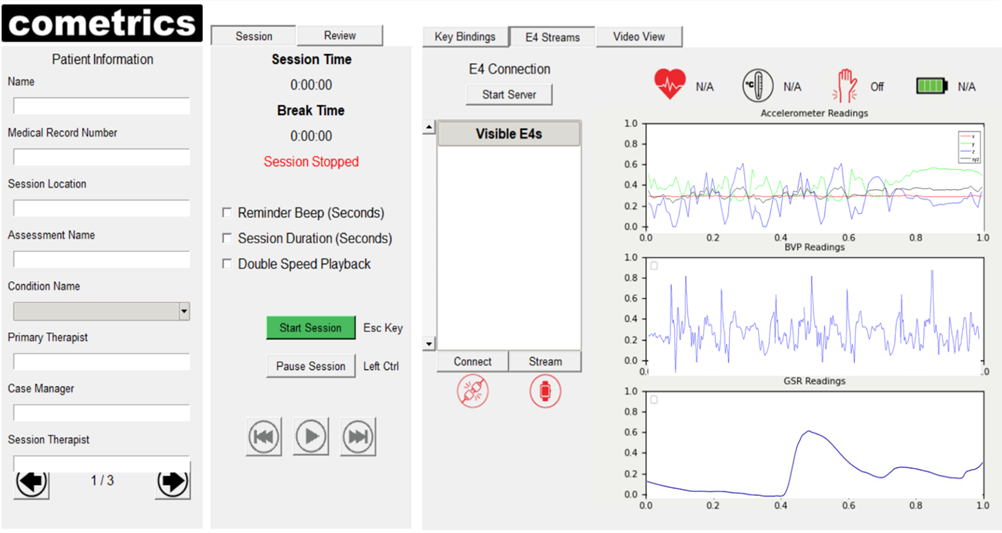


*Note.* The top display shows the key binding screen, which displays the duration and frequency keys that record the occurrence of challenging behavior or other target responses. The bottom display shows the physiological measures streamed in real-time from the E4 wristband.

Participant 1 PE Test

**Item B**

*MATLAB E4 Output File (.json) to Excel File (.xlsx) Code*

OSF data repository link: <https://osf.io/v98yb/?view_only=ee7b0d359f8a46388f671c0fd3ec9124>

**Item C**

*Example of Ledalab V3.4.9 Continuous Decomposition Analysis Across Conditions*

**
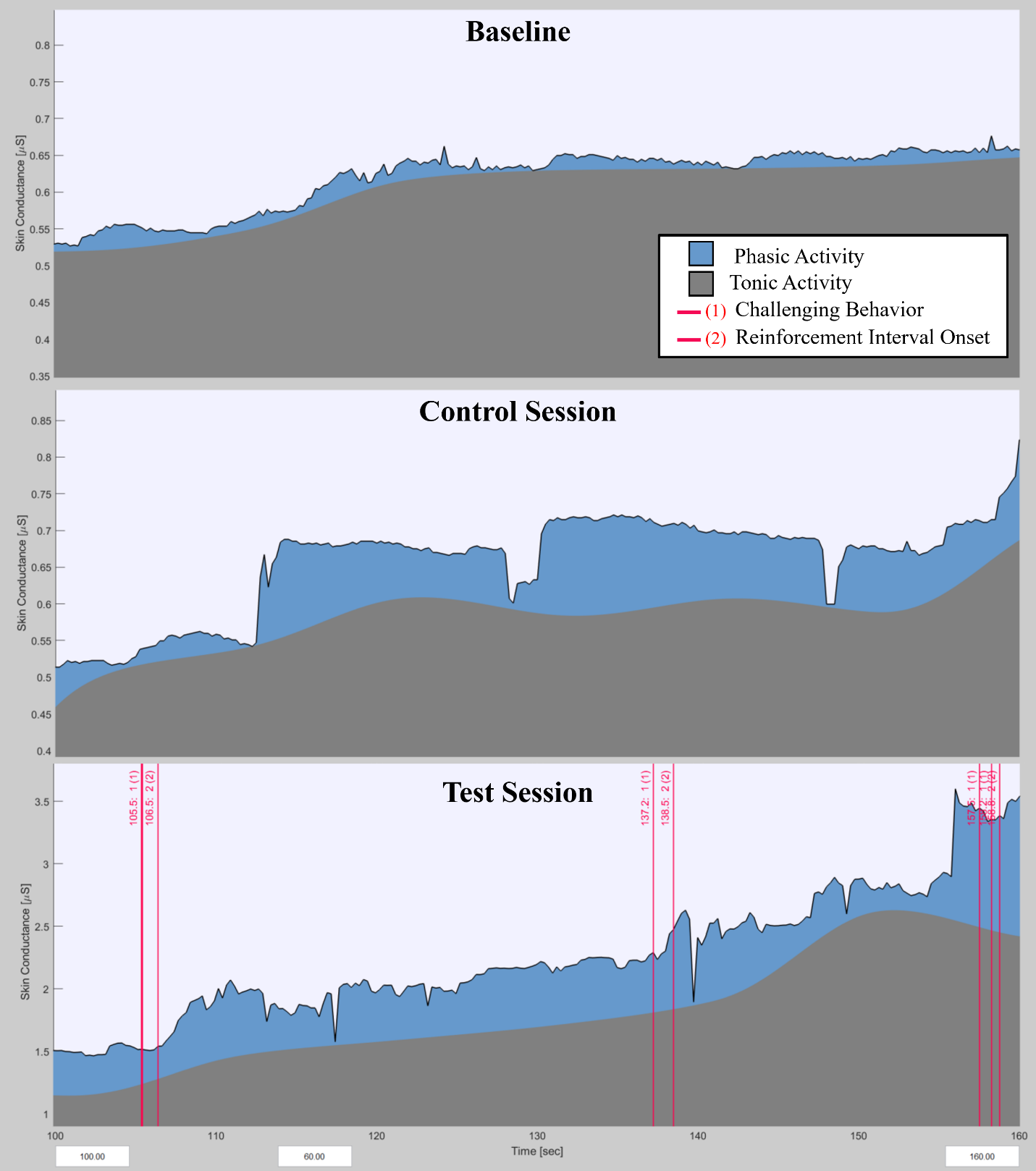
**

*Note.* µS = microsiemens. The examples depicted are from Therapist 1’s baseline (top panel), control (Session 4; middle panel), and test (Session 4; bottom panel) sessions, and represent the same time window extracted from each 10 min session.

**Item D**

*Example of Ledalab V3.4.9 Continuous Decomposition Analysis Phasic Activity Across Conditions*

*
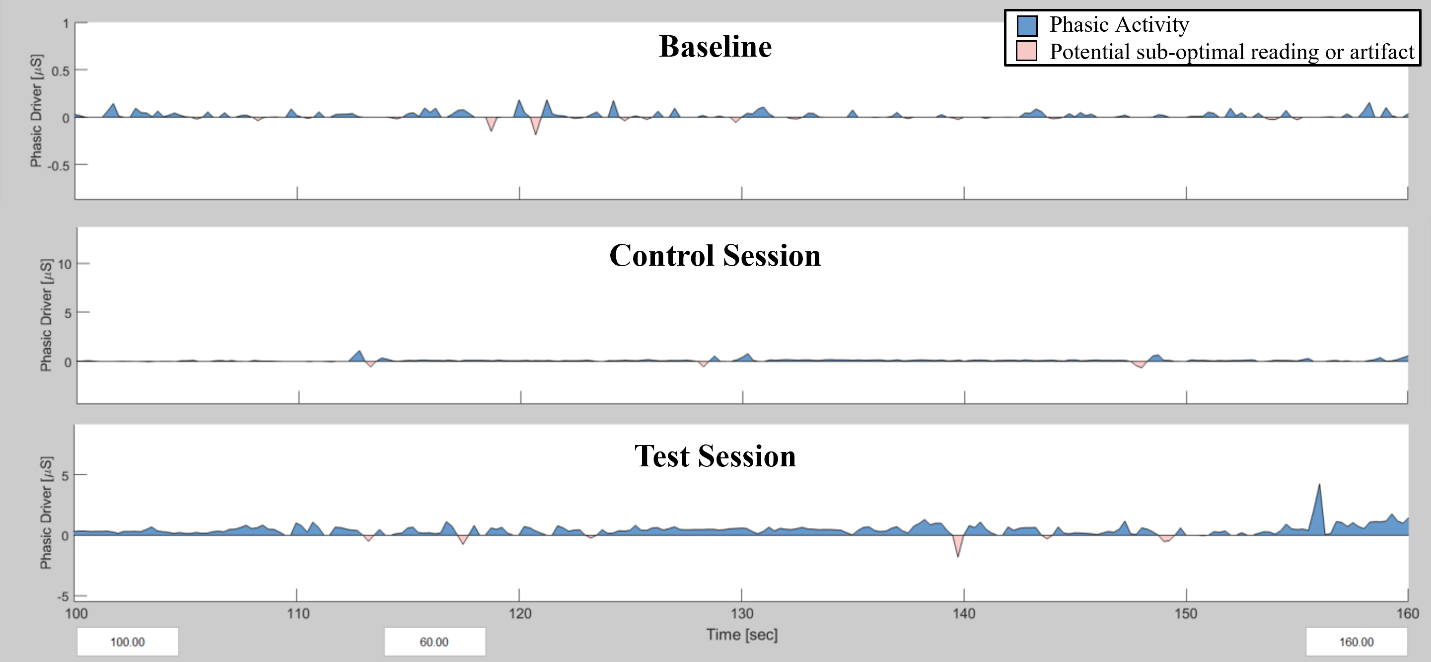
*

*Note.* µS = microsiemens. The Continuous Decomposition Analysis enables the extraction of phasic activity estimates from electrodermal activity. Displayed are the phasic activity extracted from the same time window from Therapist 1’s baseline (top panel), control (Session 4; middle panel), and test (Session 4; bottom panel) sessions. Negative values might indicate potentially suboptimal readings (i.e., producing negative values or artifacts; Bari et al., 2024), a negative SCR value, or a SCR potential that exceeds the measurement range (Wang et al., 2024).

**Item E**

*Therapist 1 Test Session 2 Graphic Example of LedaLab Continuous Decomposition Analysis*

MATLAB Excel file (.xlsx) import:


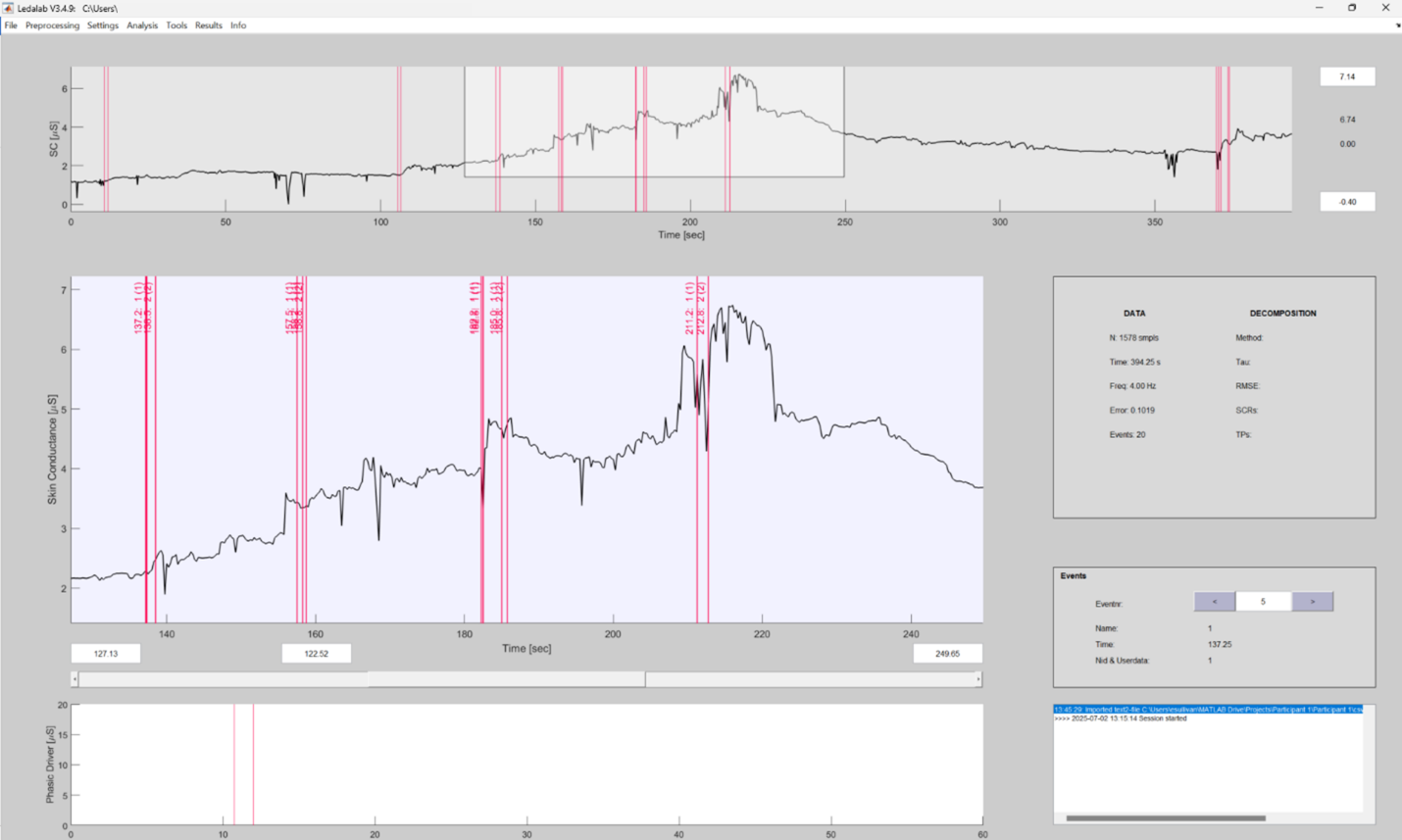


Select Continuous Decomposition Analysis and set the peak significance threshold (0.05 microsiemens [μS] in this study):

*
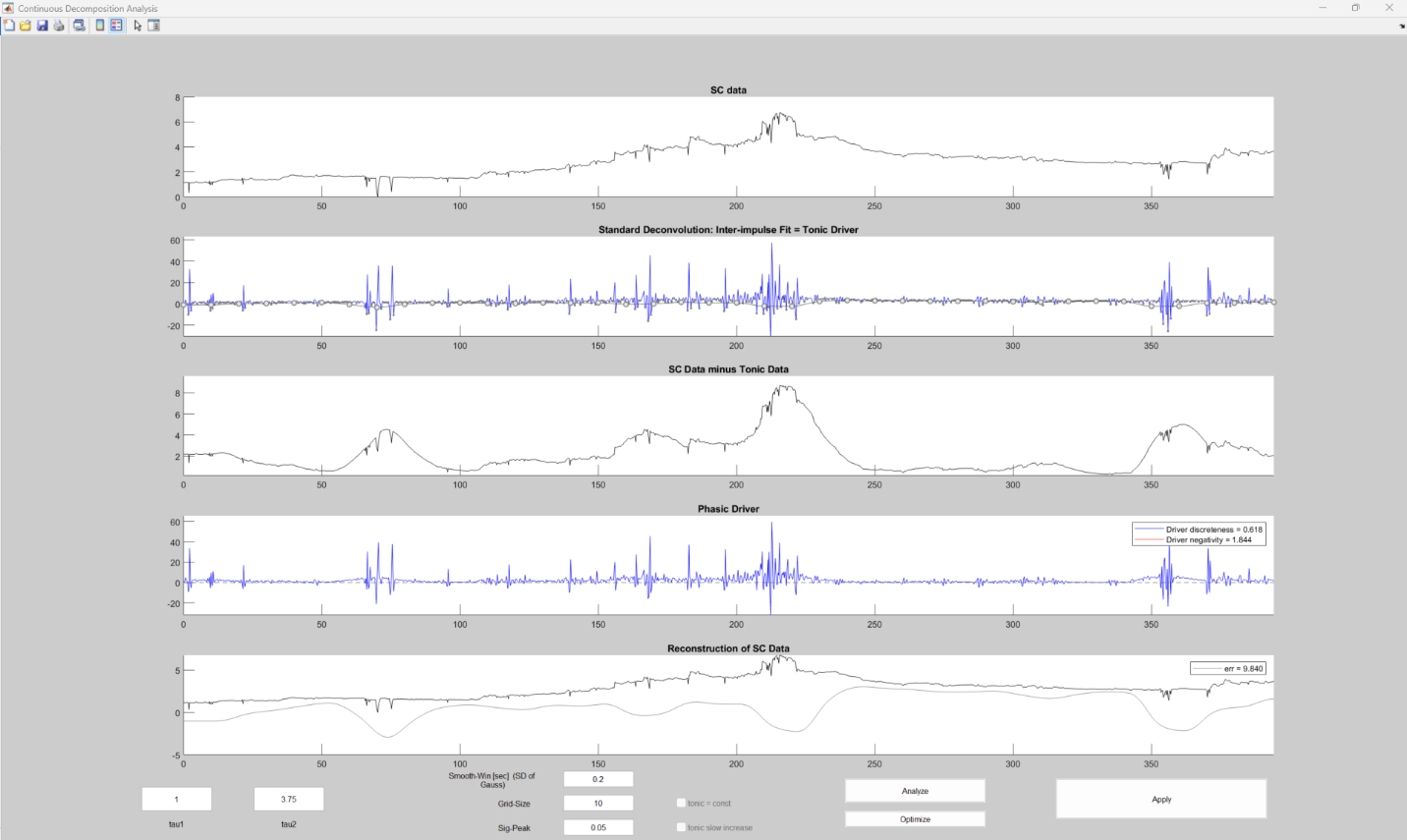
*

Apply the Continuous Decomposition Analysis and save processed data:

*
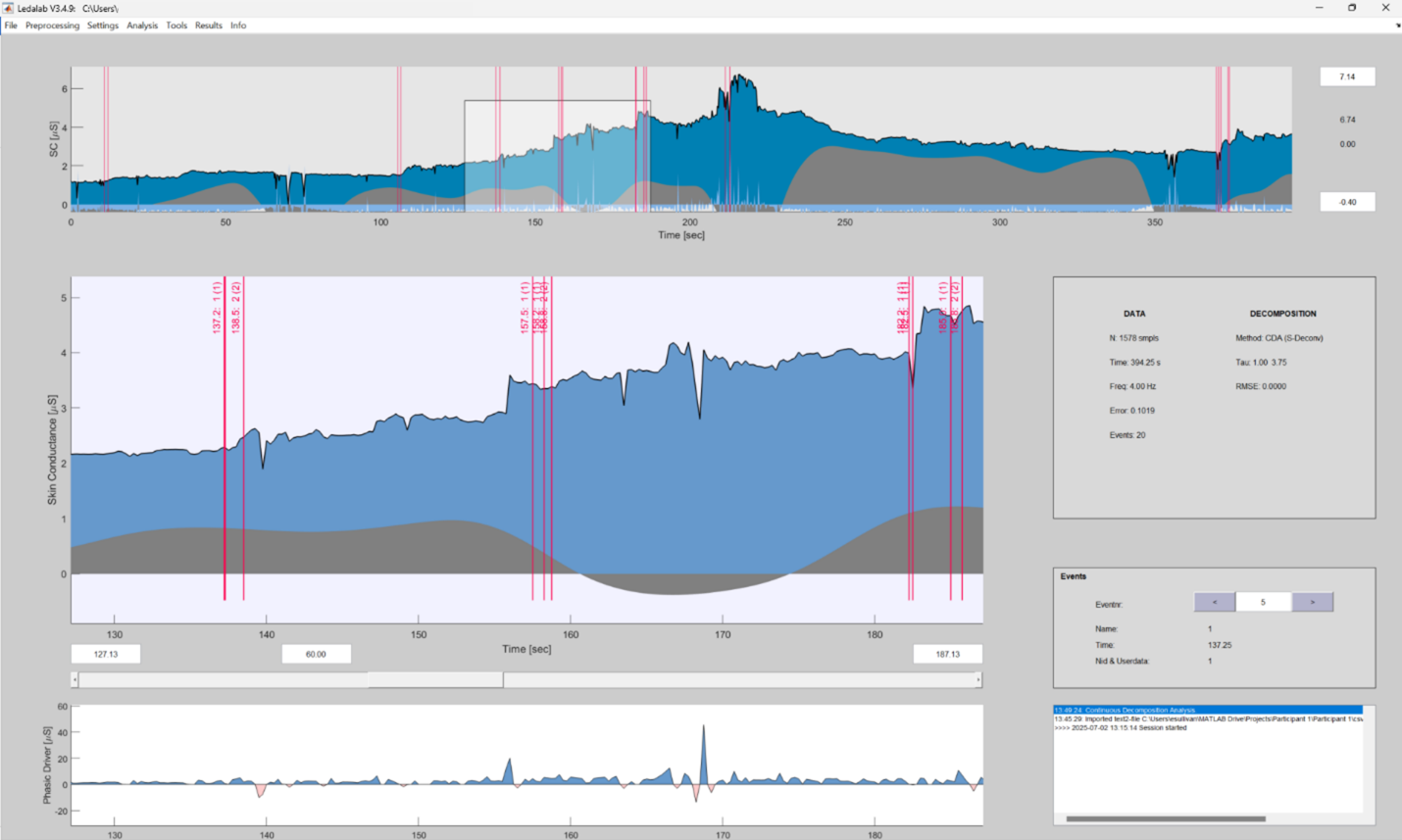
*

**Item F**

*NeuroKit2 Electrodermal Activity Output (.csv) Processing Code*

OSF data repository link: <https://osf.io/v98yb/?view_only=ee7b0d359f8a46388f671c0fd3ec9124>

**Item G**

*Therapist 2 Test Session 2 Example NeuroKit2 Processing Output Images*

*
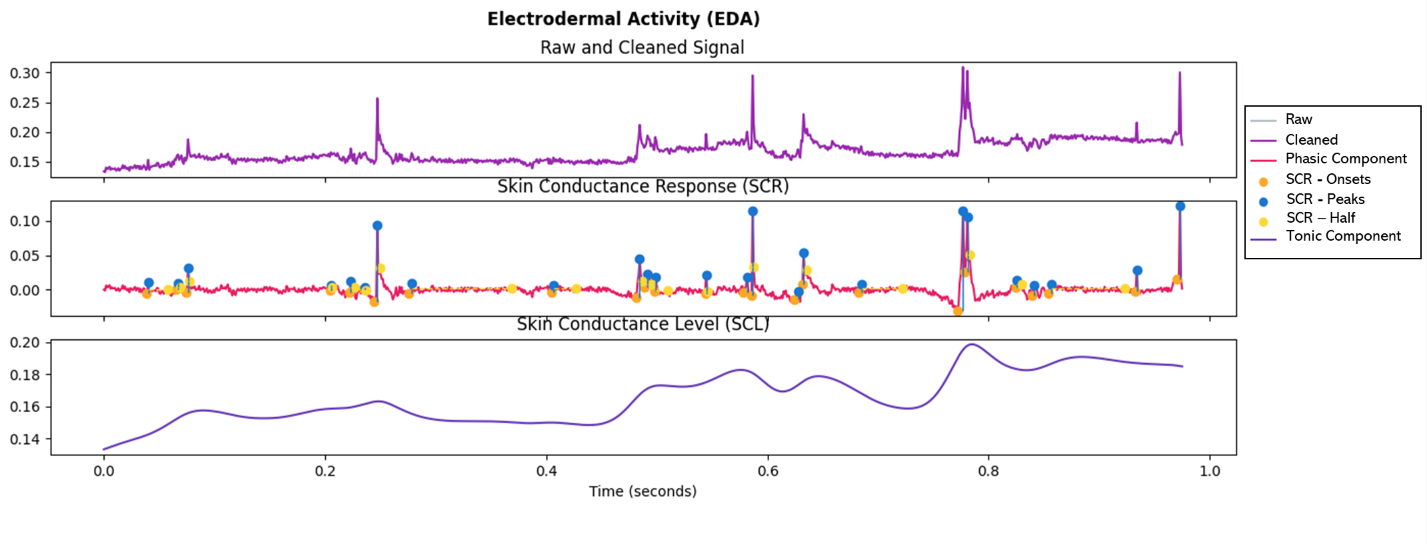
*

*
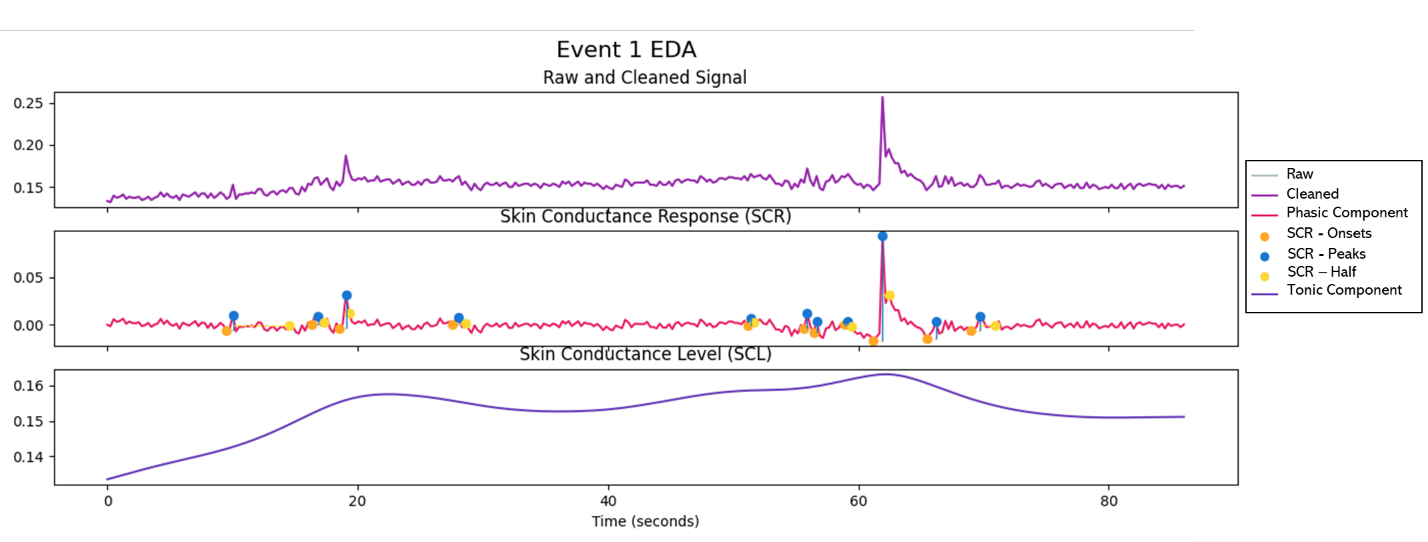
*


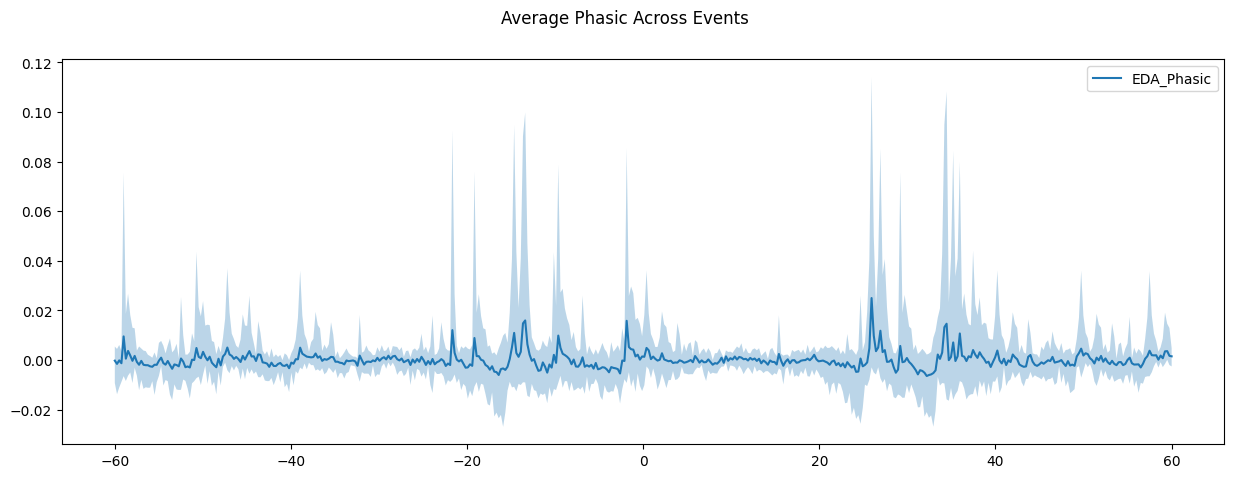


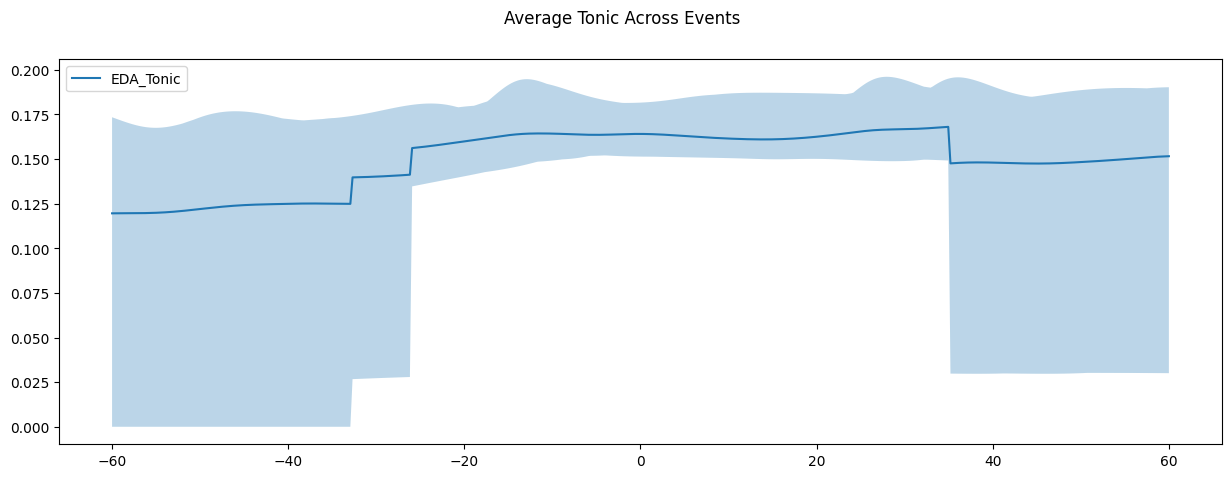


**Item H**

*Therapist 2 Test Session 2 Example NeuroKit2 Processing Output Images (additional event examples)*

OSF data repository link: <https://osf.io/v98yb/?view_only=ee7b0d359f8a46388f671c0fd3ec9124>

**Item I**

*Example of (.xlsx) File Organization of Neurokit2 Processed Data (.csv) for Ploty Graphing*

*
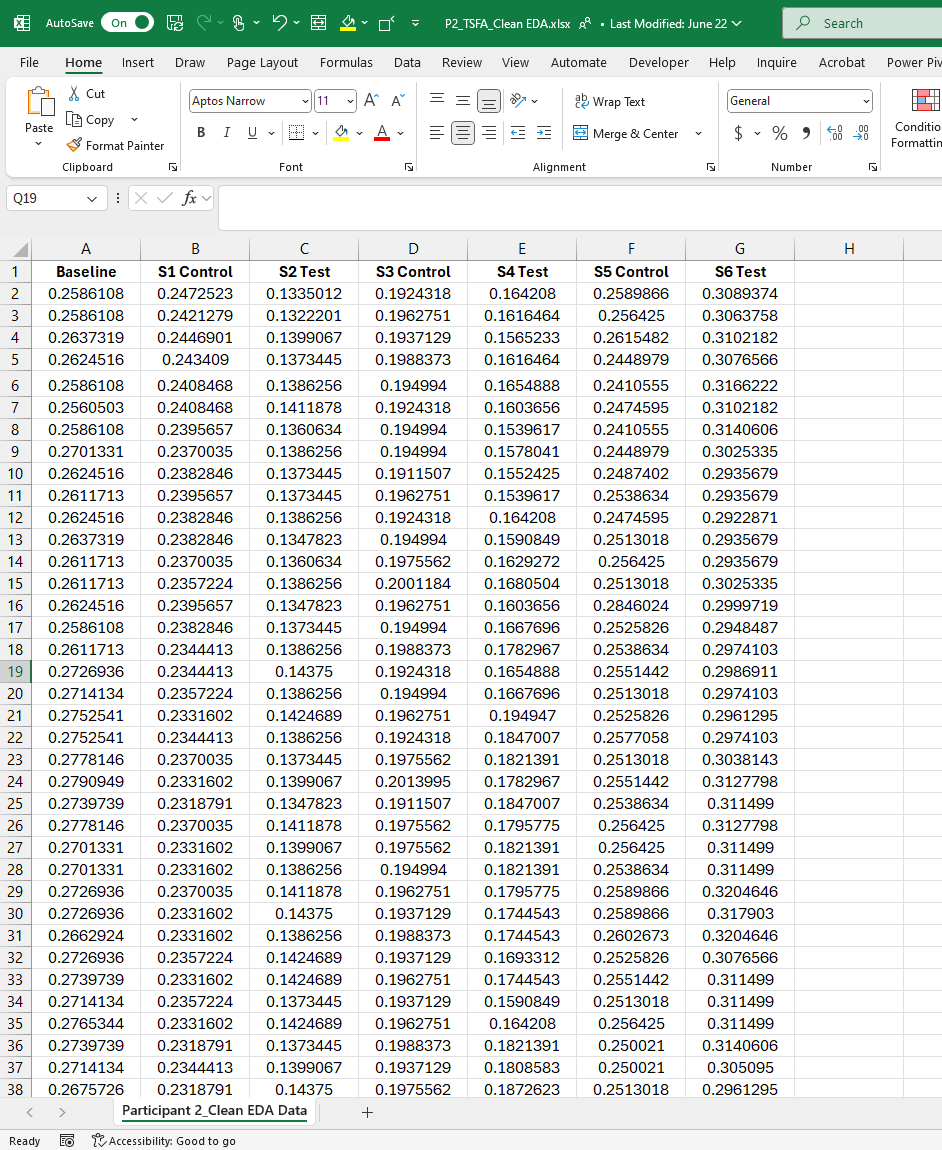
*

**Item J**

*Condition Aggregate Plotly Violin Plot from Neurokit2 Processed File (.csv) Code*

OSF data repository link: <https://osf.io/v98yb/?view_only=ee7b0d359f8a46388f671c0fd3ec9124>

**Item K**

*Therapist 1 Example Output Image of Condition Aggregate Plotly Violin Plot*


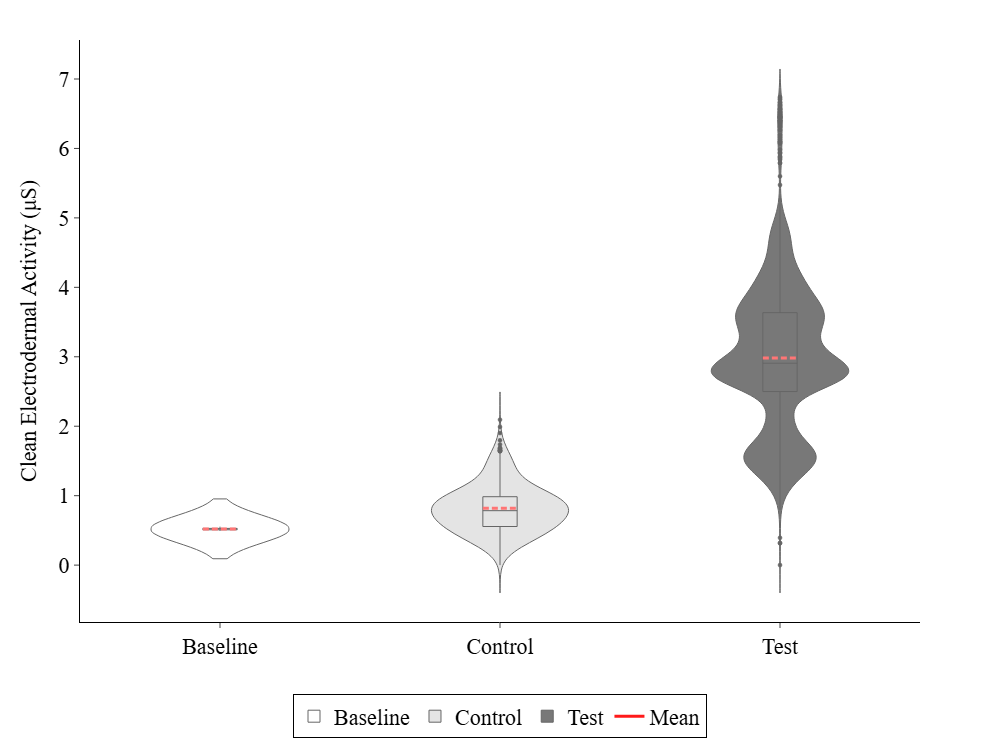


**Item L**

*Separate Conditions Plotly Violin Plot from Neurokit2 Processed File (.csv) Code*

OSF data repository link: <https://osf.io/v98yb/?view_only=ee7b0d359f8a46388f671c0fd3ec9124>

**Item M**

*Therapist Mean Clean, Phasic, and Tonic Electrodermal Activity Across Functional Analysis Sessions*

| Session | Mean Clean Electrodermal Activity | Mean Phasic Electrodermal Activity | Mean Tonic Electrodermal Activity |
| --- | --- | --- | --- |
| Therapist 1 |  |  |  |
| Baseline | 0.52 µS (0.49 µS–0.55 µS) | 0 µS (-0.02 µS–0.02 µS) | 0.52 µS (0.50 µS–0.54 µS) |
| Session 1 (control) | – | – | – |
| Session 2 (test) | 2.87 µS (0 µS–6.74 µS) | 0 µS (-1.54 µS–0.75 µS) | 2.87 µS (1.15 µS–6.16 µS) |
| Session 3 (control) | 0.72 µS (0 µS–1.13 µS) | 0 µS (-0.84 µS–0.21 µS) | 0.72 µS (0.38 µS–1.03 µS) |
| Session 4 (test) | 3.31 µS (2.45 µS–4.44 µS) | 0 µS (-1.12 µS–0.51 µS) | 3.31 µS (2.60 µS–4.09 µS) |
| Session 5 (control) | 1.13 µS (0.95 µS–2.09 µS) | 0 µS (-0.22 µS–0.63 µS) | 1.31 µS (0.98 µS–1.54 µS) |
| Session 6 (test) | 2.85 µS (2.11 µS–3.30 µS) | 0 µS (-0.65 µS–0.38 µS) | 2.85 µS (2.58 µS–2.99 µS) |
| Therapist 2 |  |  |  |
| Baseline | 0.27 µS (0.25 µS–0.42 µS) | 0 µS (-0.02 µS–0.13 µS) | 0.26 µS (0.26 µS–0.29 µS) |
| Session 1 (control) | 0.24 µS (0.23 µS–0.25 µS) | 0 µS (-0.01 µS–0.01 µS) | 0.24 µS (0.23 µS–0.25 µS) |
| Session 2 (test) | 0.17 µS (0.13 µS–0.31 µS) | 0 µS (-0.03 µS–0.12 µS) | 0.17 µS (0.13 µS–0.20 µS) |
| Session 3 (control) | 0.23 µS (0.19 µS–0.24 µS) | 0 µS (-0.01 µS–0.03 µS) | 0.21 µS (0.19 µS–0.22 µS) |
| Session 4 (test) | 0.21 µS (0 µS–0.63 µS) | 0 µS (-0.27 µS–0.34 µS) | 0.22 µS (0.17 µS–0.38 µS) |
| Session 5 (control) | 0.24 µS (0.21 µS–0.38 µS) | 0 µS (-0.02 µS–0.14 µS) | 0.24 µS (0.23 µS–0.26 µS) |
| Session 6 (test) | 0.32 µS (0.28 µS–0.47 µS) | 0 µS (-0.04 µS–0.11 µS) | 0.32 µS (0.29 µS–0.35 µS) |
| Therapist 3 |  |  |  |
| Baseline | 1.03 µS (0.99 µS–1.11 µS) | 0 µS (-0.03 µS–0.08 µS) | 1.03 µS (1 µS–1.07 µS) |
| Session 1 (control) | 2.13 µS (0.91 µS–3.61 µS) | 0 µS (-0.76 µS–0.55 µS) | 2.13 µS (0.92 µS–3.08 µS) |
| Session 2 (test) | – | – | – |
| Session 3 (control) | 0.36 µS (0.15 µS–1.13 µS) | 0 µS (-0.02 µS–0.03 µS) | 0.36 µS (0.16 µS–1.13 µS) |
| Session 4 (test) | 1.45 µS (0 µS–2.45 µS) | 0 µS (-1.84 µS–0.32 µS) | 1.40 µS (0.29 µS–2.28 µS) |
| Session 5 (control) | 0.77 µS (0 µS–2.32 µS) | 0 µS (-0.5 µS–0.37 µS) | 0.77 µS (0.30 µS–2.03 µS) |
| Session 6 (test) | 0.53 µS (0 µS–0.71 µS) | 0 µS (-0.53 µS–0.44 µS) | 0.53 µS (0 µS–0.62 µS) |

*Note.* µS = microsiemens. The values in parentheses reflect the range for each functional analysis session. Clean electrodermal activity contains both the phasic and tonic components of the recorded samples. Phasic electrodermal activity contains the fast-moving changes in autonomic system arousal, and tonic electrodermal activity contains the slower-moving changes in autonomic system arousal.

**Item N**

*Therapist Clean Electrodermal Activity Across Functional Analysis Conditions*


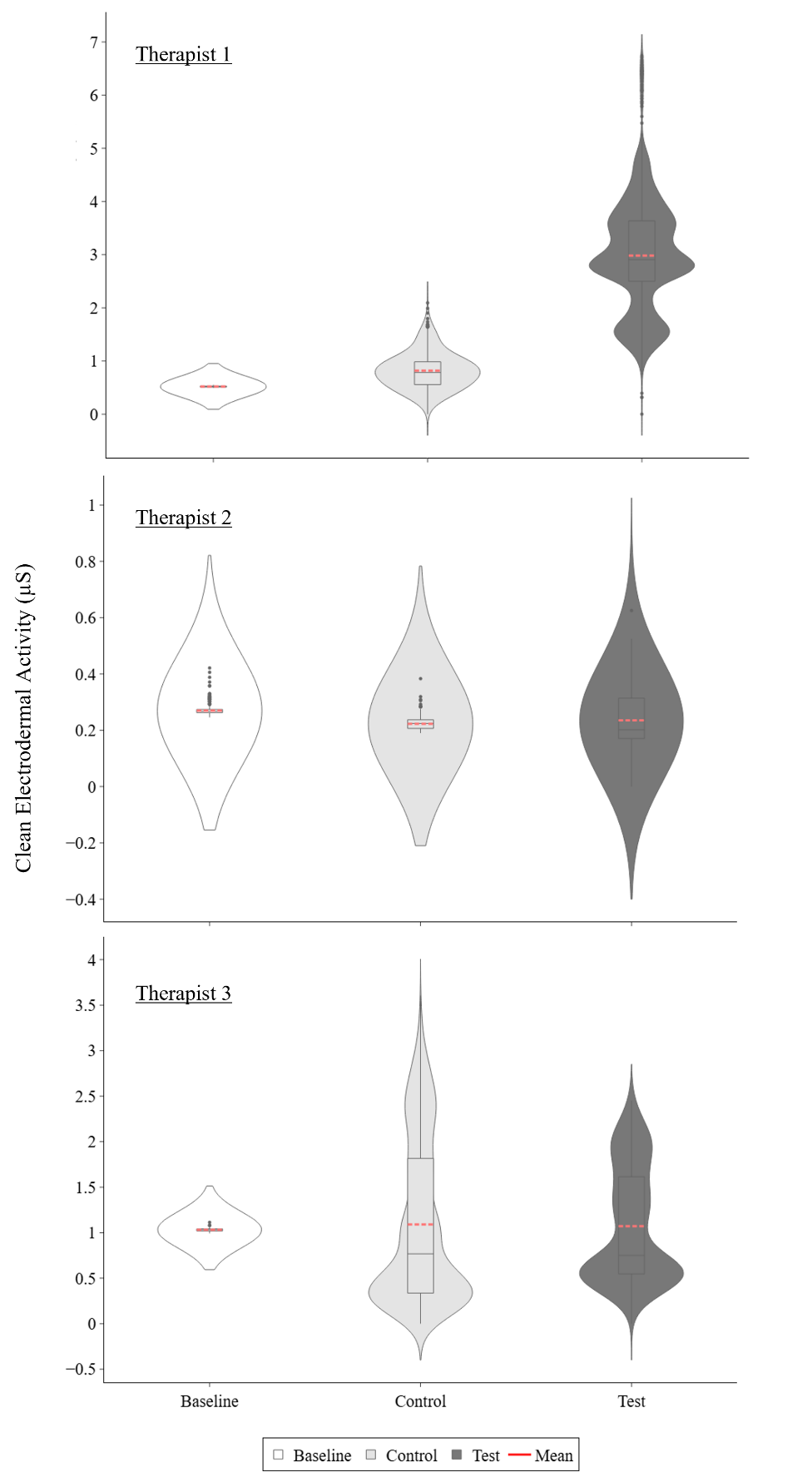


**Item O**

*Therapist 1 Example Output Image of Separate Conditions Plotly Violin Plot*

*
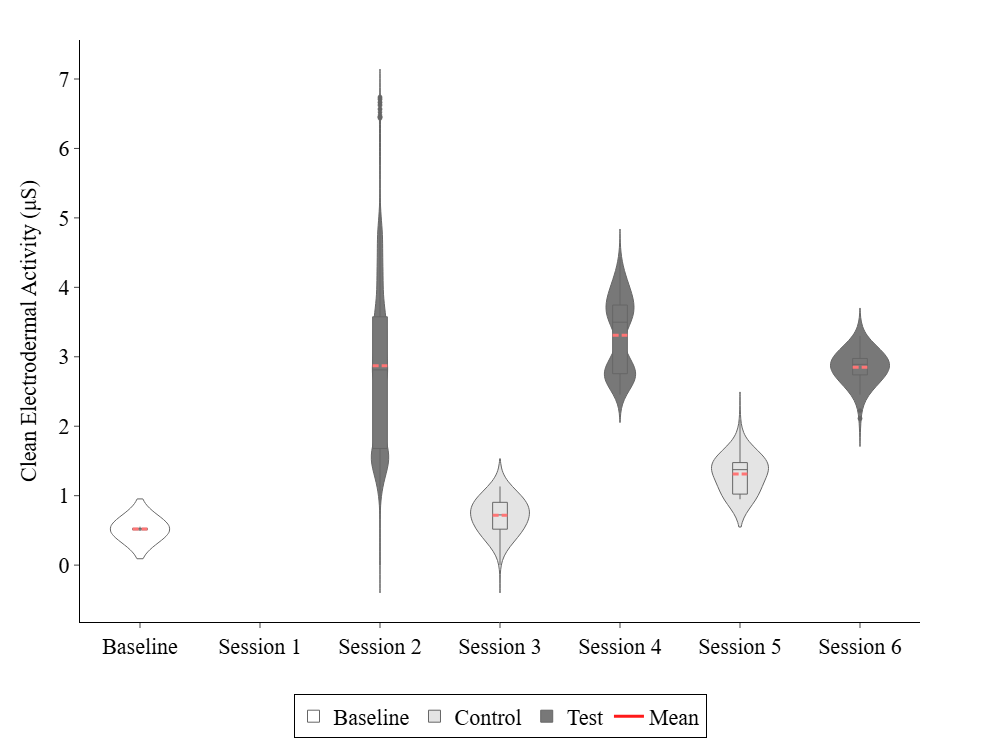
*

**Item P**

*Therapist 1 Clean, Phasic, and Tonic Electrodermal Activity Across Conditions*

*
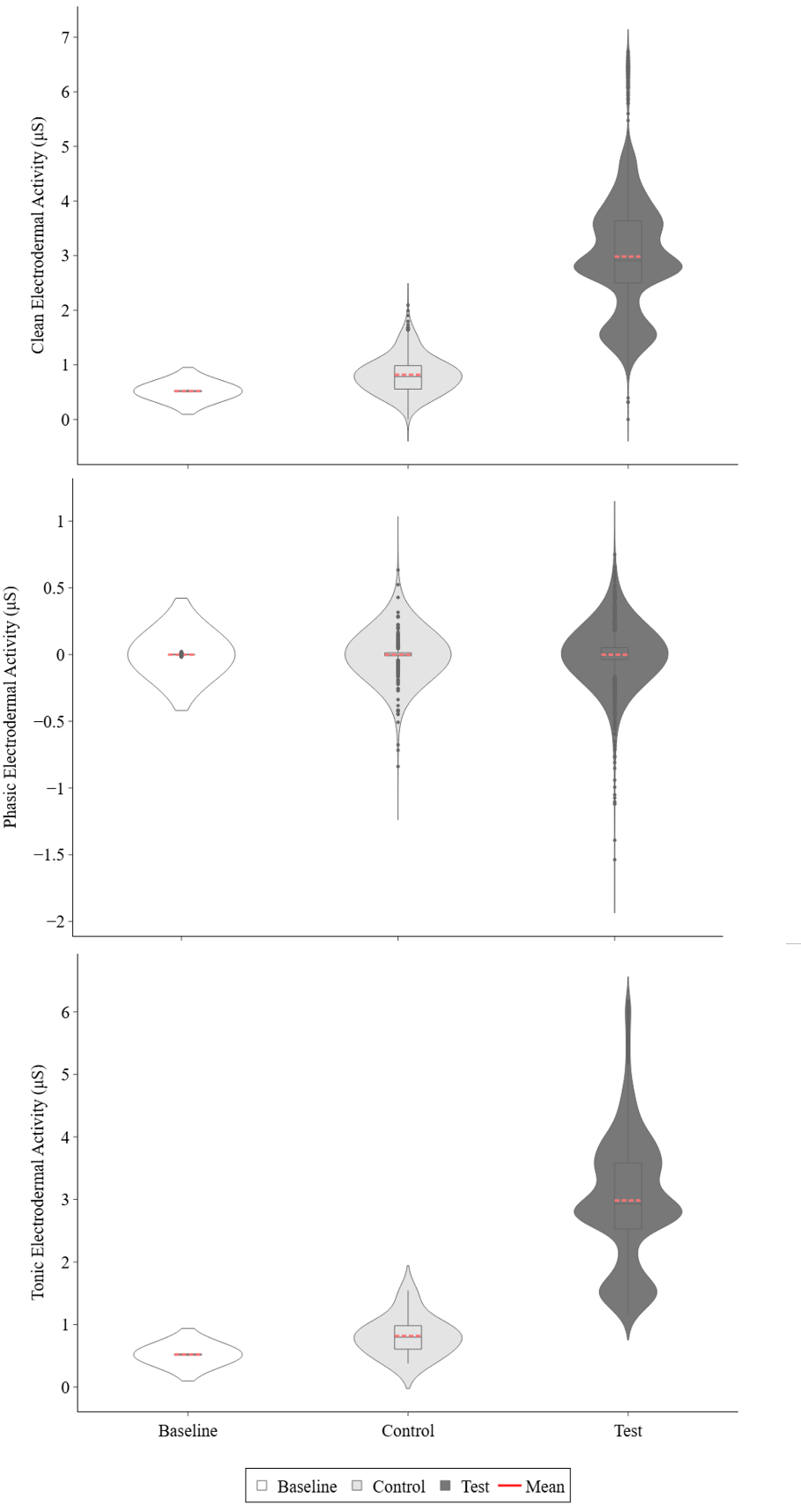
*

**Item Q**

*Therapist 2 Clean, Phasic, and Tonic Electrodermal Activity Across Conditions*

*
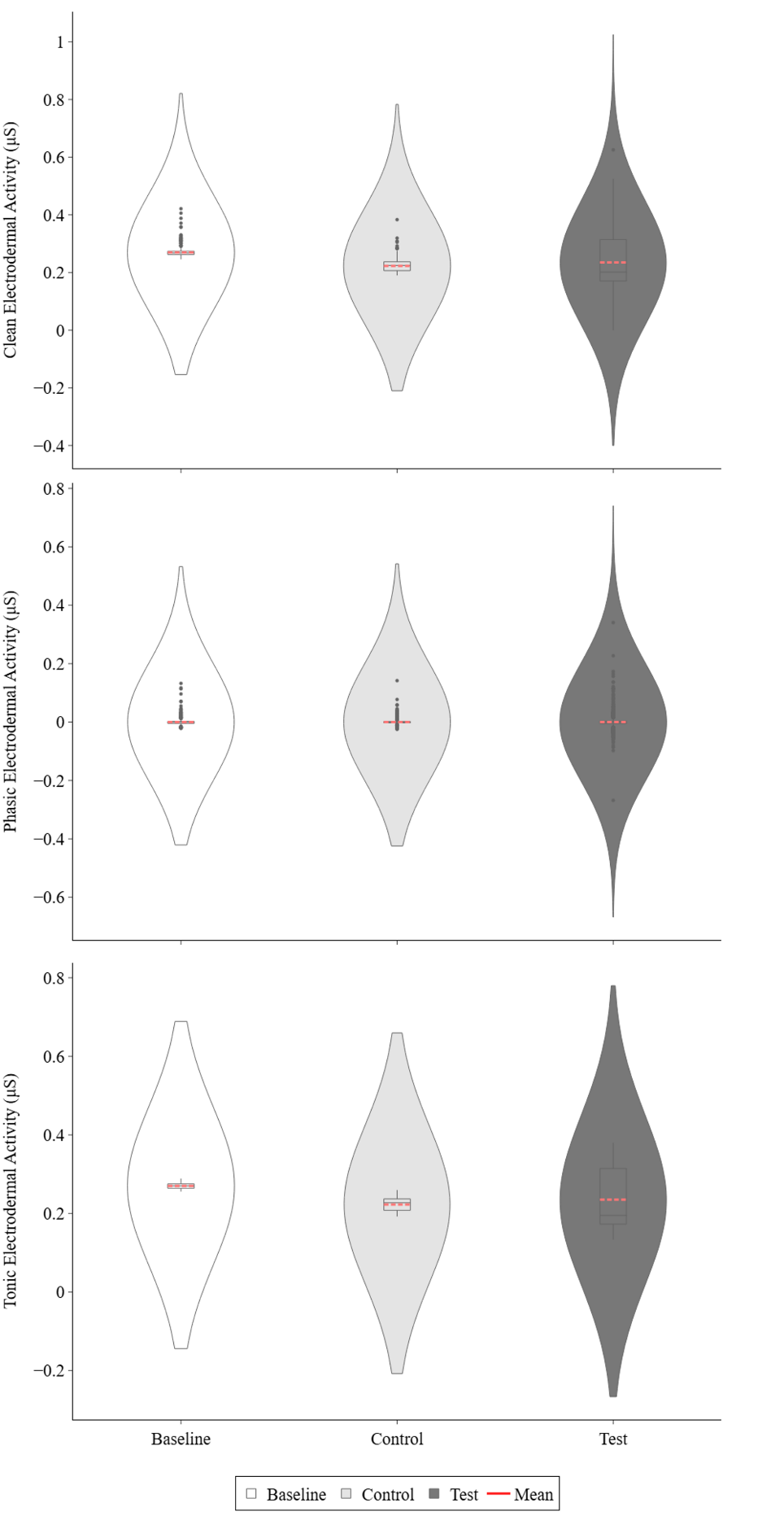
*

**Item R**

*Therapist 3 Clean, Phasic, and Tonic Electrodermal Activity Across Conditions*

*
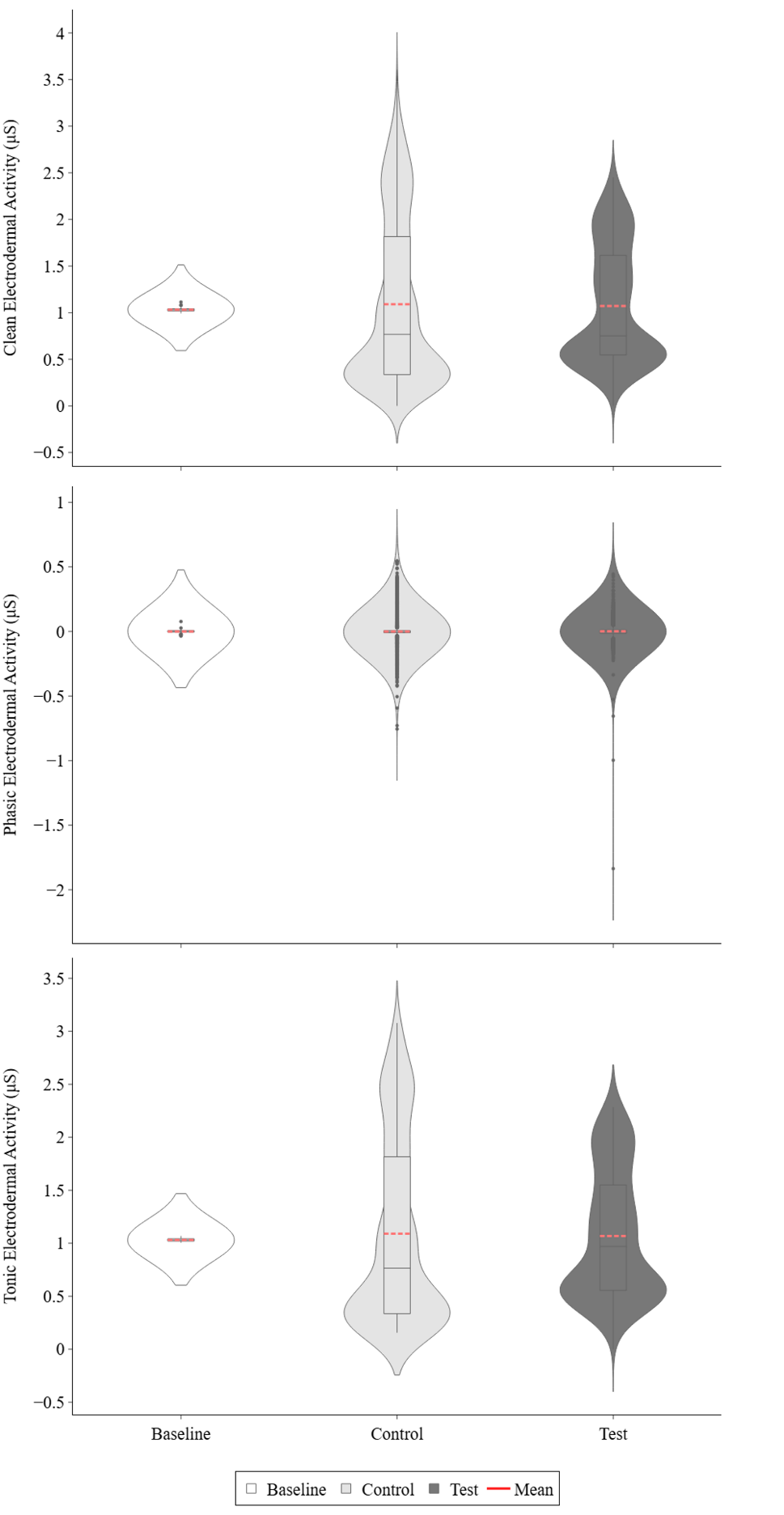
*

Raw

Cleaned

Phasic Component

SCR - Onsets

SCR - Peaks

SCR – Half Recovery

Tonic Component


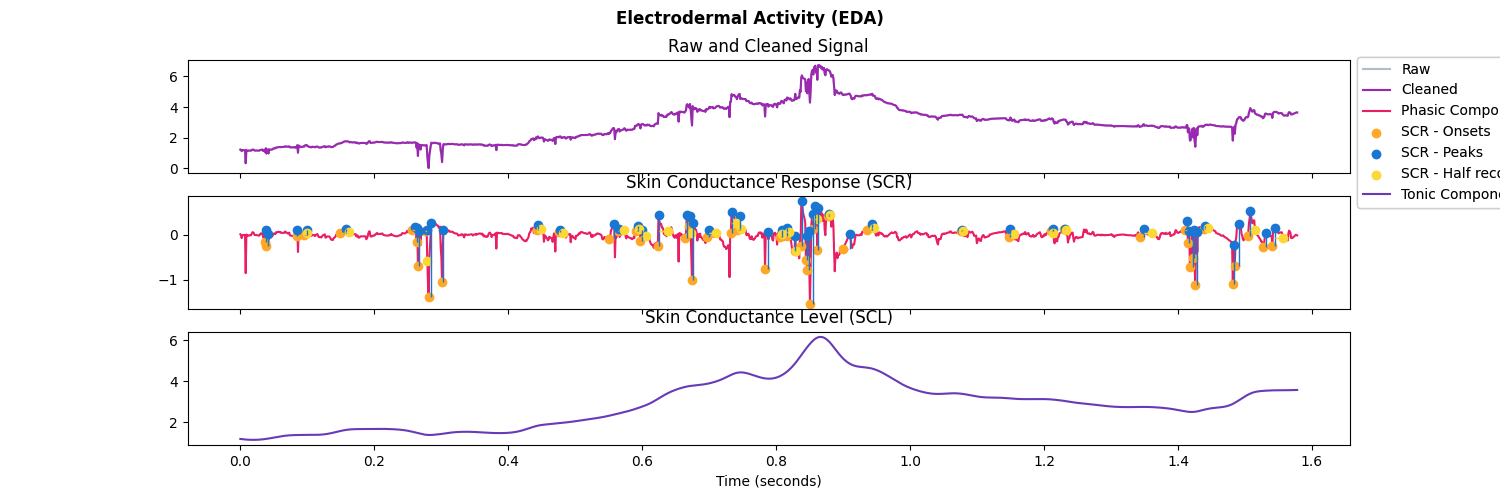

Supplement: Supplementary file 1 — Data S1 Supporting Information [file JABA-59-0-s001.docx]
